# Genomic Selection and Genome-Wide Association Analysis for Stress Response, Disease Resistance and Body Weight in European Seabass

**DOI:** 10.3390/ani12030277

**Published:** 2022-01-23

**Authors:** Stavroula Oikonomou, Athanasios Samaras, Maria Tekeoglou, Dimitrios Loukovitis, Arkadios Dimitroglou, Lefteris Kottaras, Kantham Papanna, Leonidas Papaharisis, Costas S. Tsigenopoulos, Michail Pavlidis, Dimitrios Chatziplis

**Affiliations:** 1 Laboratory of Agrobiotechnology and Inspection of Agricultural Products, Department of Agricultural Technology, School of Geotechnical Sciences, International Hellenic University, Alexander Campus, P.O. Box 141, Sindos, 57 400 Thessaloniki, Greece; mariatek55@gmail.com (M.T.); dloukovi@rias.gr (D.L.); chatz@ihu.gr (D.C.); 2 Department of Genetics, Development and Molecular Biology, Aristotle University of Thessaloniki, University Campus, 54124 Thessaloniki, Greece; 3 Institute of Marine Biology, Biotechnology and Aquaculture (IMBBC), Hellenic Centre for Marine Research (HCMR), 71003 Heraklion, Greece; tsigeno@hcmr.gr; 4 Department of Biology, University of Crete, 714 09 Heraklion, Greece; a.samaras@uoc.gr (A.S.); pavlidis@biology.uoc.gr (M.P.); 5 Research Institute of Animal Science, ELGO Demeter, Paralimni, 58100 Giannitsa, Greece; 6 Department of Research & Development, Nireus Aquaculture SA, 341 00 Chalkida, Greece; a.dimitroglou@avramar.eu (A.D.); l.kottaras@avramar.eu (L.K.); k.papanna@avramar.eu (K.P.); l.papaharisis@avramar.eu (L.P.)

**Keywords:** GWAS, stress response, disease resistance, *Vibrio*, European seabass, heritability, body weight, Dlab-chip

## Abstract

**Simple Summary:**

In the present study, the genetic architecture of the stress response, body weight, and disease resistance in European seabass were studied, providing fruitful results for further research. Initially, the above traits were recorded and genotyping on a large scale was performed in those fish. The recorded data and genotypes were combined and analyzed to find genomic areas affecting them as well as to estimate the heritability of those traits. Stress response traits and body weight were medium heritable, while genomic regions affecting them were detected. However, no genomic areas related to disease resistance were revealed. These findings improve our knowledge of the genetic structure of those traits and can be utilized in a breeding program for the genetic improvement of aquaculture broodstocks.

**Abstract:**

The majority of the genetic studies in aquaculture breeding programs focus on commercial traits such as body weight, morphology, and resistance against diseases. However, studying stress response in European seabass may contribute to the understanding of the genetic component of stress and its future use to select broodstock whose offspring may potentially be less affected by handling. A total of 865 European seabass offspring were used to measure body weight and stress response. Moreover, a disease challenge experiment with *Vibrio anguillarum* was conducted in a subset (332) of the above fish to study disease resistance. Fish were genotyped with a 57k SNP array, and a Genome-Wide Association study (GWAS) was performed. Five SNPs were found to be statistically significant, three of which affect stress indicators and body weight (in a subgroup of the population), and a putative SNP affects growth performance, while no SNP associated with resistance to *Vibrio* was found. A moderate to high genomic heritability regarding stress indicators and body weight was estimated using the Restricted Maximum Likelihood (REML) process. Finally, the accuracy, along with the correlation between Estimated Breeding Values (EBVs) and Genomic Estimated Breeding Values (GEBVs), were calculated for all the traits.

## 1. Introduction

The European seabass belongs to the Moronidae family, and apart from the wild populations naturally distributed in the North Atlantic, the Mediterranean, and the Black Sea, cultured populations are mainly farmed in Mediterranean countries. Between 1989, when the first farmed European seabass appeared, and in 2016 the production increased to 191,003 tones [1,2]. Notably, only 56% of the total production of European seabass originated from operational breeding programs using selective breeding on a limited scale, while some of the breeding programs focus only on family selection [3,4].

The main commercial traits of interest in breeding programs are growth as well as morphology and disease resistance, while broodstock selection is performed only for a small number of generations [5]. Nevertheless, apart from the traits of commercial importance, welfare traits such as stress response seem to have affected dramatically both production and disease resistance [6,7]. Although such traits have been studied in Mediterranean species, they have not yet been implemented in the breeding programs.

Stress response was studied using the cortisol, glucose, and lysozyme levels as indicators in rainbow trout (*Oncorhynchus mykiss*) as well as in Atlantic salmon (*Salmo salar*) and provided contradicting estimates on their heritability [8,9,10,11,12]. The same indicators have also been studied in Mediterranean species. The European seabass, compared to other Mediterranean fish, exhibits a high response to acute stress using cortisol, glucose, and lactate levels as bio-indicator traits [13]. Further research for post-stress cortisol levels showed that individuals of this species could be clustered into two distinctive categories showing low and high cortisol response to stress (LR and HR, respectively) [14,15]. Pinpointing on genetic parameters, studies reported a moderate heritability of cortisol levels such as 0.34 ± 0.09 to 0.37 ± 0.08 [16,17], however, earlier, Volckaert et al. [18] have reported a low heritability estimate (0.08). Moreover, a consistently negative genetic correlation was reported between cortisol levels and body weight, ranging from −0.43 to −0.60 [16,17,18]. Following the interpretation of these genetic correlations, it is evident that fish that are more easily affected by stress (HR) will have impaired growth. Furthermore, bodyweight has a range of heritability estimates from 0.38 to 0.63 [16,17,18,19,20,21]. More recently, the first approach of heritability estimation of glucose levels and lysozyme blood levels reported heritability estimates of 0.23 to 0.33 for glucose (depending on the model) and 0.56 for lysozyme levels [17].

In the European seabass, using molecular tools, such as microsatellite markers, Massault et al. [22] identified three suggestive Quantitative Trait Locus (QTL) for stress response of cortisol levels. Additionally, Chatziplis et al. [17] confirmed the above QTL in other populations and further reported one more on LG23. In the previous study, apart from QTL affecting cortisol levels, QTL affecting glucose levels have also been reported. Many studies were also performed to identify QTL for traits of commercial interest such as body weight and disease resistance [17,22,23,24,25].

Disease resistance is considered nowadays to be the second most important trait in aquaculture after growth performance [5]. A bacterial disease of high economic importance in the European seabass aquaculture is vibriosis, which is caused by various species of *Vibrio*, such as *Vibrio anguillarum* in the Eastern Mediterranean. To decrease mortality caused by vibriosis, vaccinations are needed and are widely applied [16,26]. Chatziplis et al. [17] estimated a moderate heritability of mortality after *Vibrio* injection (0.32 ± 0.07), but no evidence of any QTL affecting the trait was found.

Two new main approaches are available to improve not only genetic knowledge but also the productivity of species, Genome-Wide Association study (GWAS) and Genomic selection (GS) [27,28]. Given the recent availability of SNP arrays, GWAS can be performed to identify single nucleotide polymorphisms (SNP) associated with the phenotypic variation of the traits of interest [29]. The detected QTL can then be used in Marker Assisted Selection (MAS) to improve the selection process of the candidates [30]. An example of the beneficial use of MAS had been observed in the Atlantic salmon with the resistance against Infectious pancreatic necrosis (IPN). When a major QTL (on chr 26) was reported to affect the outcome of the IPN, it was used to select the breeding candidates via MAS; because of such selection, fish deaths from IPN were close to zero [28].

The identification of a significant locus is important in the above described process selection (i.e., MAS), but it can be considered as the main limitation as well. Focusing on Genomic selection, all the available genomic information is considered to estimate the relationship among all individuals, independently of the individual effect of genomic variation to the phenotype. An advantage of using GS is that it is more effective when the phenotypic variation of a trait is affected by multiple genomic regions [27,28,31,32].

The current study aims to (i) identify SNP associated with bodyweight at different growth stages, fish welfare (in the form of stress bio-indicators), and disease resistance, and (ii) estimate heritability and breeding values using a classic pedigree-based polygenic model as well as genomic selection for the aforementioned traits.

## 2. Materials and Methods

### 2.1. Ethical Statement

All experiments were performed in accordance with relevant guidelines and regulations. Nireus S.A. research facilities are certified and have obtained the codes for the rearing and use of fish for scientific purposes (EL04-BIOexp-01). All procedures on fish used in this study were approved by the Departmental Animal Care Committee following the Three Rs principle, in accordance with Greek (PD 56/2013) and EU (Directive 63/2010) legislation on the care and use of experimental animals.

### 2.2. Population

In total, 865 offspring and their 110 parents (broodstock) have been utilized in this study, originating from two breeding periods (referred to as batches 10 and 13). The sample consisted of 14 full-sib and 68 half-sib families in both batches (Pedigree information was available only for one generation, and more information about population structure and offspring per family is available in Supplementary File S1, Table S1). Specifically, 533 offspring were reared in experimental tanks from February 2014 to March 2014 (batch 10), and 332 offspring were reared in experimental tanks from August 2018 to October 2018 (batch 13). Fish were individually PIT-tagged in both batches. In batch 10, each family was separately reared in one 140 liter (l) circular tank (62 tanks), whereas, in batch 13, each 140 l tank included one offspring from each family (20 tanks). Throughout both experimental periods, the photoperiod was set at 12L:12D, the water temperature was 18.20 ± 0.05 °C, and the salinity 27. Oxygen and pH ranged between 6–10 mg L−1 and 7.20–7.40, respectively.

### 2.3. Study Design and Measurements

In both batches, a stress test was performed three consecutive times (once per month) as described by Pottinger and Carrick [33,34] and modified by Fanouraki et al. [13] and Samaras et al. [14]. Briefly, fish were chased in the tank for 5 min with a net and confined for 30 min at 1/3 of the tank’s initial volume. After each stress test, fish were anesthetized in 300 phenoxyethanol, and blood samples were collected from the caudal vessel using heparinized syringes, and body weight was measured at the following ages: 318–334 Days Post Hatching (DPH), 346–362 DPH, and 362–378 DPH. Apart from the previous measurements, body weight was also measured in 290–306 DPH.

Blood samples were used to measure the plasma concentration of cortisol, lysozyme, lactate, and glucose levels. Cortisol, glucose, and lysozyme levels were determined using the methods described by Chatziplis et al. [17]. Lactate levels were determined by commercial enzymatic colorimetric assays (Spinreact, Girona, Spain).

Furthermore, seven months after the stress test experiment and a resting period of one week, 332 offspring (out of the total 533 offspring of batch 10) were placed in the same tank and injected with 0.1 mL of 3.36 × 103 cfu mL^−1^ *Vibrio anguillarum* and mortality was measured as a binary trait (Binary survival, Dead/Alive, more details concerning the *Vibrio* challenge in Chatziplis et al. [17]). The experiment duration was 11 days.

### 2.4. Genotyping and Quality Control

Fish and their respective breeders were genotyped with the Thermo Fisher Axiom TM Seabass 57k SNP Dlab-Chip [24] at the genotyping platform Gentyane (INRAE, Clermont-Ferrand, France) using DNA isolated from fin-clip tissue. 56,730 SNPs were filtered using the following parameters: SNPs call rate lower than 90%, minor allele frequency lower than 0.05, and deviation of the Hardy–Weinberg equilibrium *p* < 0.001. Moreover, individuals were filtered based on a sample call rate lower than 90% and genotype similarity of more than 98%. Out of the total number of SNPs and individuals, 50,136 and 972 (862 offspring and their 110 parents) have been used for further analysis, respectively. For quality control, plink software was used [35].

### 2.5. Estimation of Heritability

#### 2.5.1. Stress Indicators

Heritability of the stress indicators was estimated using Restricted maximum likelihood procedure (REML). A univariate mixed animal model with repeated measurements for each stress indicator was used to estimate the heritability and the repeatability of the traits. One fixed effect with 2 levels was fitted in the model since two batches from different seasoning were utilized. Furthermore, the polygenic component was fitted in the model using Pedigree Relationship Matrix (PRM) (Equation (1)).

(1) Y=Χb+Ζυ+Wpe+e

where *Y* corresponds to the vector of repeated measurements per stress indicator, *b* is the fixed effect (batch), *X*, *Z*, and *W* are incidence matrices that related observation to fixed, random, and permanent environmental effects. The *υ* is the additive genetic effect using a PRM, and it is described as ~*N* (0, *Aσ_a_*^2^) (*A* is the PRM and *σ_a_*^2^ is the additive variance), and the *pe* is the permanent environment effect. Finally, *e* is the random residual.

A repeatability model without any relationship matrix per stress indicator was used to calculate the corrected phenotypes based on the repeated measurements per offspring described by Åkesson et al. [36], however, it was modified as no fixed effect was used. This analysis was performed using BLUPF90 [37], and the generated solutions are referred to as corrected phenotypes for each stress indicator, and they were used as phenotypes in a univariate animal model (Equation (2)) for the estimation of the heritability (so permanent environment (*pe*) was not included in Equation (2), as it did in Equation (1), since corrected phenotypes were used and were estimated using the repeatability model without any relationship matrix). In Equation (2), one fixed effect with 2 levels was fitted as well as the polygenic component was estimated using Genomic Relationship Matrix (GRM).

(2) Y=Χb+Ζυ+e

where *Y* corresponds to the corrected phenotypes of the stress indicators, *b* is the fixed effect, *X* and *Z* are incidence matrices that related observation to fixed and random effects, *υ* is the additive genetic effect using a GRM, and it is described as *~N (0*, *Gσ_a_^2^)* (*G* is the GRM and *σ*_a_^2^ is the additive variance). Finally, *e* is the random residual. The heritability of Equation (1) was compared to the genomic heritability of Equation (2) for each stress indicator.

#### 2.5.2. Body Weight

The heritability of the body weight, as well as the genetic parameters between measurements, were estimated using REML. In terms of body weight, the four measurements were used in a multitrait animal model to estimate heritability and genetic/phenotypic correlations among them. The batch was used as a fixed effect, while the polygenic effect, which was fitted in the model, was estimated using two approaches, fitting the PRM and GRM (Equation (2)). In Equation (2), *Y* corresponds to the matrix of the body weight measurements.

The estimation of heritability of all the traits (Equations (1) and (2)) and the genetic/phenotypic parameters for growth were performed using AIREMLF90 [37,38]. The heritability was estimated using the additive genetic variance divided by the total phenotypic variance (the sum of additive genetic variance and residual variance) [h^2^ = *σ_a_*^2^/(*σ_a_*^2^ + *σ_e_*^2^)] for body weight and corrected phenotypes for stress response. While for repeated measurements of the stress response the variance of permanent environment was also included in the total phenotypic variance [h^2^ = *σ_a_*^2^/(*σ_a_*^2^ + *σ_e_*^2^ *+* *σ_pe_*^2^)] and repeatability was estimated by the sum of the additive genetic variance and the permanent environment variance divided by the total phenotypic variance [r = (*σ_a_*^2^ + *σ_pe_*^2^)/(*σ_a_*^2^ + *σ_e_*^2^ *+* *σ_pe_*^2^)]. The calculation of the corrected phenotypes was performed using BLUPF90 [37], and the created solutions of this model were used to estimate the heritability of the stress indicators (Equation (2)) using the AIREMLF90 [37,38].

### 2.6. GWAS Analysis

#### 2.6.1. Stress Indicators

A univariate linear mixed model with repeated measurements GWAS for each stress indicator (cortisol, glucose, lactate and lysozyme levels, Equation (3)) was performed in RepeatABEL/R software [39,40]. In the model, the batch was used as a fixed effect.

(3) Y=Χ b+Z SNP+υ+W pe+e

where *Y* corresponds to the vector of repeated measurements per stress indicator, *b* is the vector of the fixed effect (batch), *Z* is the indicator the matrix of the tested SNP, the *SNP* is the effect of the tested SNP, *X* and *W* are incidence matrices that related observation to fixed and permanent environmental effects. The *υ* is the additive genetic effect using the GRM, and it is described as ~*N* (0, *Gσ_a_*^2^) (*G* is the GRM and *σ_a_*^2^ is the additive variance), and the *pe* is the permanent environment effect. Finally, *e* is the random residual.

Furthermore, a univariate linear mixed model was performed per measurement per trait as well as on corrected phenotypes of the stress indicators (Equation (4)) using GEMMA software (version 0.94.1) [41] fitting the batch as a fixed effect.

(4) Y=Χ b+Z SNP+υ+e

In Equation (4), the *Y* corresponds to the vector of each measurement per stress indicator or the corrected phenotypes of each stress indicator, *b* is the fixed effect (batch), *Z* is the indicator of the matrix of the tested SNP, the *SNP* is the effect of the tested SNP and *X* is the incidence matrix that related observation to fixed effects. The *υ* is the additive genetic effect using the GRM, and it is described as ~*N* (0, *Gσ_a_*^2^) (*G* is the GRM and *σ_a_*^2^ is the additive variance). Finally, *e* is the random residual.

#### 2.6.2. Body Weight

A multitrait linear mixed model was used to predict the association between SNPs and growth performance. The four measurements of the body weight were used as dependent variables in the model (Equation (4)). Thus, the *Y* corresponds to the matrix of the body weight measurements in Equation (4). GWAS was performed using GEMMA software [42].

In the multitrait GWAS, offspring were tested not to deviate from multivariate normality using the Mahalanobis distance, as described by Shim et al. [43]. Offspring with a *p*-value less than 0.01 were considered as outliers and removed from the analysis. The Mahalanobis distance estimation was performed in R [44].

Furthermore, a univariate linear mixed model per measurement was also performed using GEMMA software (version 0.94.1) [41], and thus, the *Y* corresponds to a vector containing each measurement of the body weight in Equation (4).

#### 2.6.3. Disease Resistance

A univariate linear mixed model was used to identify the association between SNP and disease resistance against *Vibrio anguillarum*. Mortality was used as a dependent variable, described as binary data (Dead/Alive) in the model. GWAS was performed using GEMMA software [41]. There was no fixed effect (batch) in this analysis as all fish were from the same batch (a subgroup of batch 10).

The Bonferroni correction method was used to minimize the false-positive results in all of the above models [45], and it was applied at the genomic level using the 0.05 and 0.1 as levels of significance (the corresponding threshold after the Bonferroni correction was estimated using the 0.05/total number of SNPs or 0.1/total number of SNPs). Finally, the proportion of phenotypic variance (PVE) explained by a given SNP was estimated as described by Shim et al. [43] to estimate the SNP effect on each trait (Equation (5)).

PVE = (2 b^2^ MAF (1-MAF))/[2 b^2^ MAF (1-MAF) + (se (b))^2^ 2 N MAF (1-MAF)] (5)

where the *MAF* is the Minor Allele Frequency of the SNP, the *b* is the effect size estimated, and the *N* is the sample size.

The sequence of the significant SNP from GWAS were cross-referenced against the European seabass genome database to identify any linked genes that could be used as possible candidates (http://seabass.mpipz.mpg.de, accessed on 15 July 2021).

### 2.7. Genomic Prediction and Comparison with Pedigree-Based Approach

For the estimation of the breeding values for stress indicators as well as for the body weight, the Best Linear Unbiased Prediction (BLUP) approach was used [46]. For the genomic selection, the Genomic Best Linear Unbiased Prediction (GBLUP) approach [31,32] was used to estimate the Genomic Estimated Breeding Values (GEBVs) for those traits, while Estimated Breeding Values (EBVs) were estimated using the BLUP approach fitting the PRM, to compare both approaches [47]. Breeding values from all the models in Section 2.5 were estimated using PREGSF90 and BLUPF90 [37,38]. For the stress indicators, the Equation (1) model was used to estimate the EBVs, and the Equation (2) model was used to estimate the GEBVs. For the body weight, the Equation (2) model (as it was modified in Section 2.5.2) was used in both models fitting the PRM and GRM, for EBVs and GEBVs, respectively.

Furthermore, the accuracy of the methods was estimated using five replicates of a fivefold cross-validation analysis, with the training set including 80% and the validation set including 20% of the population. In the validation set, all the offspring were selected randomly, and their phenotypes were masked to estimate accuracy [48]. The phenotypes for body weight and stress indicators (corrected phenotypes and repeated measurements) were also corrected for the fixed effect. The correlation coefficient between corrected phenotypes for the fixed effect and breeding values in the masked offspring was estimated, and it was divided by the square root of the heritability for each model and trait. For the stress indicators, the square root of the heritability of Equation (1) for the pedigree-based approach was used, while for the genomic selection approach, the square root of the heritability of the Equation (2) model was fitted to compare the accuracy of each approach. On the other hand, for the body weight, only one model was used (Equation (2)) to estimate heritability. The square root of the heritability used the PRM for the pedigree-based approach, while for the genomic selection approach, the square root of the heritability using the GRM was fitted to estimate the accuracy of each approach. Furthermore, for all the traits, the accuracy for GEBVs was also estimated using the correlation between GEBVs and corrected phenotypes divided by the square root of the heritability estimated by BLUP (using the PRM) [48,49].

Finally, the correlation between EBVs (using PRM) and GEBVs (using GRM) was calculated using the Spearman method for the body weight and stress indicators [50]. The correlations were calculated in R [44].

## 3. Results

### 3.1. SNPs after QC

The descriptive statistics of all traits are illustrated per batch as well as jointly in Table 1. Out of the total number of SNPs, 44,691 were distributed on 24 chromosomes, while the remaining 5445 could not be assigned to a chromosome and were included on one chromosome named 25 (Supplementary File S1, Table S3 illustrates the corresponding LGs). Based on the karyotype of the European seabass, there are 24 chromosomes [51], thus, chromosome 25 includes the unlinked SNPs. Chromosomes 5 and 17 included the highest and lowest number of SNPs in comparison with the rest of the chromosomes, 2353 and 1014, respectively.

### 3.2. Estimation of Heritability and Genetic Parameters

#### 3.2.1. Stress Indicators

Using the PRM, the heritability of cortisol, glucose, lactate, and lysozyme levels under a repeatability animal model was estimated at 0.45, 0.31, 0.61, and 0.63, respectively (Table 2). The repeatability of the cortisol, glucose, lactate, and lysozyme levels was estimated at 0.48, 0.32, 0.62, and 0.64, respectively. When the GRM was used in the genetic analysis, the heritability of cortisol, glucose, and lactate levels were 0.43, 0.52, and 0.59, respectively. Finally, the heritability of lysozyme levels was 0.75 (Table 2).

#### 3.2.2. Body Weight

Estimates of heritability of body weight in different ages during the experiment ranged from 0.54 to 0.75, using the PRM. Using the GRM, the heritability of weight per measurement ranged from 0.54 to 0.61 (Table 3 and Table 4). The genetic correlation between measurements ranged between 0.87 and 0.99 when using the PRM and between 0.83 and 0.99 when using the GRM. While the phenotypic correlation ranged from 0.81 to 0.98 (Table 3 and Table 4).

### 3.3. GWAS

#### 3.3.1. Stress Indicators

**Figure 1:**
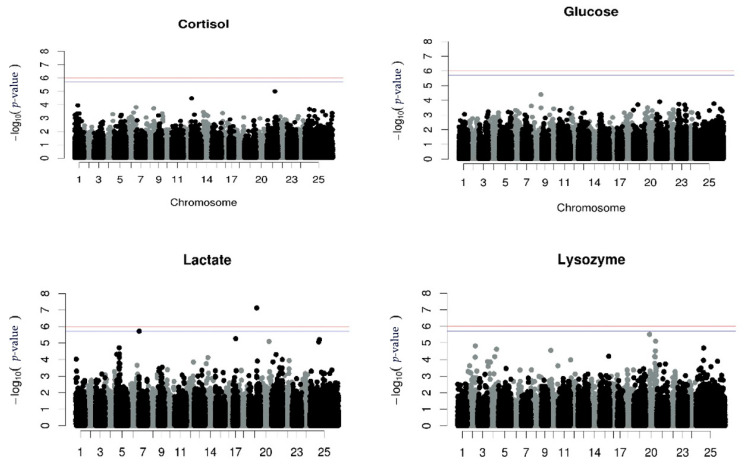
Manhattan plots per trait using GWAS with repeated measurements. The red (initial value 0.05) and the blue line (initial value 0.1) illustrate the threshold after Bonferroni correction (at the genomic level).

No significant association between SNPs and cortisol, glucose or lysozyme levels was detected using the repeated measurements GWAS analysis. However, one SNP associated with lactate levels in chromosome 19 (LG5) was found. The proportion of the phenotypic variance which was explained was 2.6% (Figure 1, Table 5).

Manhattan plots for univariate GWAS for each measurement of all the stress indicators and the univariate GWAS for the corrected phenotype for the stress indicators are illustrated in Supplementary File S2 (Supplementary File S2, Figures S2–S5 and Figures S6–S9, respectively). The results from the repeated measurements using the RepeatABEL/R [39,40] were identical to the results from corrected phenotypes using the GEMMA (version 0.94.1) [46] software. Focusing on those GWAS, there was one SNP associated with the first measurement of lysozyme levels, on chromosome 20 (LG6), and the proportion of phenotypic variance explained by the SNP was 2.2% (Supplementary File S2, Figure S5b), while two SNPs associated with the third measurement of lactate levels in chromosomes 17 and 19 (LG3 and LG5, respectively) were revealed, explaining 2.5% of the phenotype each (Supplementary File Supplementary File S2, Figure S4d).

Analyzing batch 10, the QTL affecting lactate levels at the 3rd measurement located in chromosome 19 (LG5) was also detected to be significant in the present subgroup, with an approximately similar effect (PVE, 2.3%, Table 6).

#### 3.3.2. Body Weight and Disease Resistance

**Figure 2:**
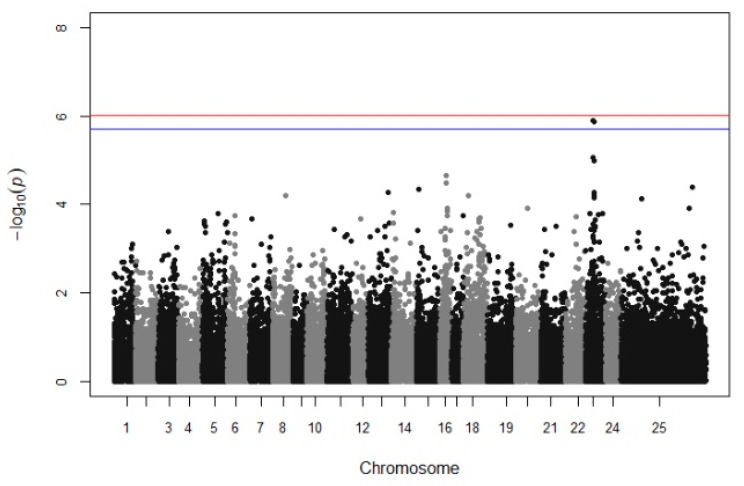
Manhattan plots for the multivariate GWAS of body weight. The red (initial value 0.05) and the blue line (initial value 0.1) illustrate the threshold after Bonferroni correction (at the genomic level).

A suggestive QTL was found on chromosome 23, providing a sufficient trailing using multitrait GWAS in batches 10 and 13 (Figure 2). However, when each growth stage was tested individually, no significant association was detected. Manhattan plots from univariate GWAS for the body weight at each growth stage and per batch are illustrated in Supplementary File S2 (Supplementary File S2, Figures S1 and S10).

Focusing on univariate GWAS for batch 10, two SNPs were associated with the second, third, and fourth measurements of body weight on chromosome 16 (LG24) when only batch 10 was analyzed (Supplementary File S2, Figure S10). The proportion of phenotypic variance (PVE) for those SNPs ranged from 2.4% to 2.7% and from 2.5% to 2.9% (Table 6).

**Figure 3:**
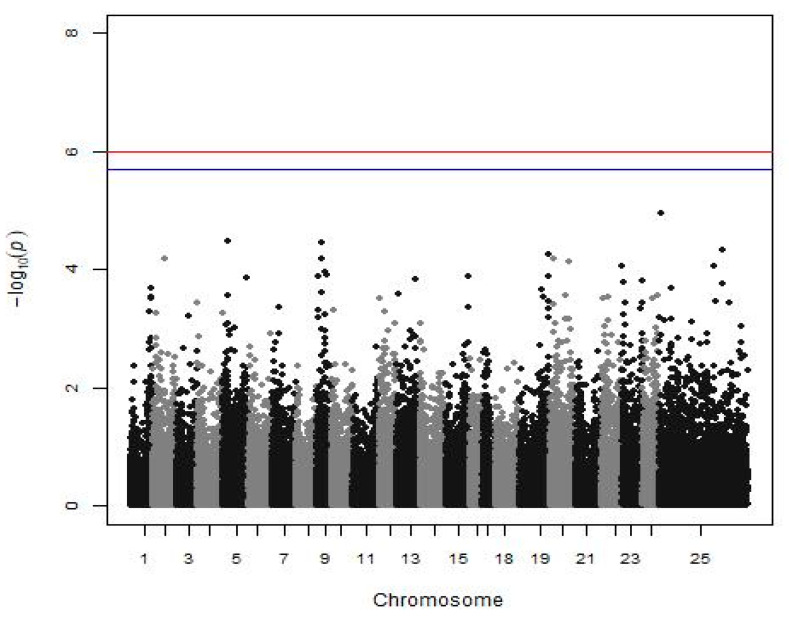
Manhattan plot shows the results of disease resistance against *Vibrio* (a subgroup of batch 10). The red (initial value 0.05) and the blue line (initial value 0.1) illustrate the threshold after the Bonferroni correction (at the genomic level).

Analyzing the batch 10 subgroup for disease resistance, 42.8% of the fish were dead until the 11th day of the experiment, and no significant association between SNP and disease resistance against *Vibrio* was found (Figure 3).

The sequences of the significant SNP from GWAS were cross-referenced against the European seabass genome database, and the possible candidates of genes are illustrated in Supplementary File 1, Table S4 (http://seabass.mpipz.mpg.de, accessed on 15 July 2021). The SNP (AX-172322981) affecting body weight was close to the *fgf14* gene, based on Itoh and Ornitz [52], who reported that the *Fibroblast Growth Factor* (*FGF*) family gene is involved in multiple biological procedures; however, *fgf14* affects the neurological functions. Finally, none of those genes were related to the traits that are studied presently.

### 3.4. Genomic Selection and Comparison with Pedigree-Based Approach

For stress indicators, the accuracy of EBVs was between 0.26 and 0.47 using the PRM, while for GEBVs ranged from 0.28 to 0.51 (Table 7). Cortisol and lactate levels showed higher accuracy prediction when using genomic relationship matrix than when using the pedigree relationship matrix, while lysozyme levels showed similar estimates. On the other hand, higher accuracy using PRM was detected for glucose levels. Additionally, comparing the accuracy between the PRM and the GRM with $\surd h_{PRM}^{²}$ (instead of the square root of heritability of the GBLUP), the latter approach showed higher estimation for the cortisol, lactate, and lysozyme levels, equal to 0.48, 0.28, and 0.48, respectively.

In terms of body weight, the accuracy of EBVs was between 0.42 and 0.54 for all growth stages using the PRM, while for GEBVs ranged from 0.40 to 0.60 (Table 7). The last two measurements of the body weight showed a slightly raised estimation using the pedigree relationship matrix compared to the genomic relationship matrix. In addition, the accuracy with GRM using $\surd h_{PRM}^{²}$, provided higher estimations in all the measurements of the body weight compared to the accuracy estimated with the PRM (Table 7).

Correlations between EBVs and GEBVs for biochemical and hormonal stress indicators ranged from 0.78 to 0.84 for all phenotypes. Finally, correlations between EBVs and GEBVs for body weight ranged from 0.85 to 0.91 for all measurements (Table 8).

## 4. Discussion

Broodstock and their offspring from two breeding seasons (batches) underwent genetic analysis using the 57k SNP Dlab-chip array [24] and GWAS was performed using all the trait measurements on: (i) growth, (ii) stress performance using hormonal (cortisol), biochemical (glucose, lactate) and immunological (lysozyme) biomarkers, and iii) mortality after *Vibrio anguillarum* injection. Furthermore, the heritability of the traits mentioned above was estimated, while for body weight, the genetic parameters were also estimated using PRM and GRM. Genomic selection and pedigree approaches were applied for all the traits. Finally, the correlation between breeding values as well as accuracy was calculated for stress indicators and growth.

### 4.1. Heritability of the Stress Indicators

Generally, cortisol levels showed a contradicted heritability among species because, in Atlantic salmon, they were low (0.07 and 0.05) [8,12], while in rainbow trout, they ranged from low to high (0.17 to 0.50) [8,9,10,34]. In terms of glucose, both species showed low heritability (Atlantic salmon 0.03 and rainbow trout 0.07) [8]. Finally, the heritability of lysozyme levels in Atlantic salmon was 0.19 [12], while in rainbow trout, it was 0.32 [9,10]. In European seabass, the heritability of cortisol levels was reported as 0.35–0.34 in most studies [16,17] and 0.08 [18,22]. The heritability of lysozyme was 0.55 and of glucose levels was low (0.23, 0.33) [17].

In our study, the heritability of cortisol and lactate was similar regardless of the use of the PRM and the GRM, indicating that genomic and pedigree-based relationships are providing similar estimates in a polygenic inheritance model. Our estimates of cortisol levels were similar to the estimates for the rainbow trout than those for the Atlantic salmon, while comparing them with the estimates for the European seabass, they are higher than the reported range (0.08–0.34) [16,17,18,22].

However, the heritability of the glucose and lysozyme levels showed a different pattern to that of cortisol and lactate levels. The heritability of lysozyme levels was higher in the model using the GRM than the PRM, providing an increase of 0.12 in the estimate. Even though there was no significant QTL affecting it using GWAS with repeated measurements, one SNP associated with the first measurements of lysozyme levels was detected using univariate GWAS. The findings mentioned above could explain this increase. Chatziplis et al. [17] estimated the heritability of lysozyme levels lower than the present study (0.55) using a subgroup of our population (batch 10).

Finally, the heritability of glucose levels showed a higher increase, jumping from 0.32 to 0.52 using the GRM. However, none of the performed GWAS showed any significant SNP-phenotype associations on the results, and the findings remained low, without any significant peaks in all the Manhattan plots. The heritability estimated by Chatziplis et al. [17] was similar to the present heritability using the PRM and generally higher than the reported estimates for the Atlantic salmon and rainbow trout.

### 4.2. Heritability and Genetic Parameters for Body Weight

The heritability of body weight in European seabass ranges from 0.38 to 0.63 in different growth stages (illustrated as 179 to 397 DPH or 29.5 to 741.9 gr) using pedigree relationship matrix [16,18,19,20]. Apart from body weight in European seabass, the heritability of the growth performance had been estimated to be 0.39 (PRM) and 0.76 (GRM) [49]. In Dupont-Nivet et al. [19], the heritability was 0.46, using fish from four different farms analyzed jointly, but independently, it ranged from 0.38 (516.6 gr) to 0.44 (336.6 gr), when the maternal effect was fitted in the model a drop of 0.11 was noticed (h^2^ = 0.36).

In this study, the heritability ranged from 0.75 to 0.54 (weight 1 to weight 4) using the PRM, while it was estimated as 0.61 to 0.57 using the GRM. Compared to the estimates in the literature, the heritability of weights 3 and 4 were in the reported range, but the heritability of weights 1 and 2 were higher than it. In our case, a drop of 0.14 in the heritability of weight 1, as well as a 0.1 decrease in weight 2, was noticed when the GRM was used. Weight 3 and 4 showed a similarity between the estimates, apart from an increase of 0.03 in the latter. However, Besson et al. [49] reported an increased heritability when using the GRM compared to when using the PRM, studying the growth for the European seabass (almost double). The high genetic correlation between the measurements remained similar regardless of the type (PRM or GRM) of the relationship matrix used in the analyses.

Finally, body weight is affected by the stress response, and a negative genetic correlation between cortisol levels and body weight has been reported in different populations by Chatziplis et al. [17] and Vandeputte et al. [16]. In the present study, a negative genetic correlation was also found when using the GRM instead of the PRM [−0.46 (0.11)], which was statistically significant, while none of the rest indicators were shown to affect the growth (data not shown).

### 4.3. GWAS of Stress Indicators

A few studies have reported stress response as a heritable trait, and QTL on linkage groups 1, 3, 14, and 23, using microsatellite markers, which explained a range of 0.15% to 10% of the proportion of phenotypic variation of stress response have been identified [17,22]. In contrast, the results from the present study showed no statistically significant association between cortisol levels and any SNP.

Starting with cortisol levels, the QTL reported by Chatziplis et al. [17] and Massault et al. [22] correspond to chromosomes 5, 11, and 17, but using the 57K Array [24], no trailing appears in those chromosomes. Focusing on glucose and lysozyme levels, Chatziplis et al. [17] identified QTL using microsatellite markers corresponding to chromosomes 5 and 17, but in the present study using association analysis, no statistically significant QTL was detected as well as no trailing appears in those chromosomes. The only increased trailing appears on chromosome 20 for lysozyme levels using repeated measurements (and corrected phenotypes). However, using only the first measurement of lysozyme levels in univariate GWAS, the same trailing appears, providing a statistically significant QTL (Supplementary Material S2, Figure S5b). Finally, for lactate levels, a significant QTL was detected on chr 19 when using the repeated measurements and the 3rd measurement, however, this QTL was not significant when the 1st and 2nd measurements were analyzed independently (Supplementary File, Figure S4)

### 4.4. GWAS of Body Weight

QTL affecting the body weight has been reported on different LGs [17,22,23] using microsatellite markers in European seabass. Focusing on, Chatziplis et al. [17] used a subgroup of our population (batch 10), reported QTL affecting body weight in LGs 1, 3, 4, 6, and 14, which corresponded to chromosomes 11, 17, 25, 20, and 5, respectively. However, in our case, no significant QTL was detected when univariate and multivariate GWAs were performed in those chromosomes.

Focusing on the increasing trailing in chr 23 (LG9), there is evidence of a genomic region that affects the growth performance because firstly, two SNP are above the threshold 0.1 (with Bonferroni correction), and secondly, there is also a trailing of SNP associations appearing in Manhattan plot (Figure 3). However, when univariate GWAS was used, no trailing appeared in chr 23 (LG9). Generally, the use of multitrait GWAs can increase the power of QTL detection since multiple measurements were combined and used in the same analysis, compared to a univariate GWAS [53]. An example of the increased statistical power was attributed by Yoshida et al. [53], who used multi GWAS and the detection power was raised from 13% to 44%, contrary to using a univariate GWAS. Meanwhile, in univariate GWAS analysis, two SNP were identified to be associated with body weight in the 2nd (318–334 DPH), 3rd (346–362 DPH), and 4th (362–378 DPH) measurement when only batch 10 was analyzed (chr 16, Supplementary File S2, Figure S10b–d). However, there was no evidence of a significant association between the previous SNP in the same area or any other area when batch 13 was analyzed. This could be explained due to the lower average body weight and/or the smaller sample size of batch 13 (332 fish).

### 4.5. GWAS for Disease Resistance

QTL affecting disease resistance against *Vibrio anguillarum* have been found in other species, such as the rainbow trout, the Japanese flounder (*Paralichthys olivaceus*), and the half-smooth tongue sole (*Cynoglossus semilaevis*) [54,55,56,57]. Focusing on the European seabass, Chatziplis et al. [17] used a subgroup of our population (batch 10), and the estimated heritability of disease resistance against *Vibrio* injection at 0.32 ± 0.07, thus mortality appears to be a heritable trait. However, no QTL linked with microsatellite markers was detected in that study [17] or in our analysis in search of a significant association between mortality after *Vibrio* injection and SNPs (Figure 1). Nevertheless, the power of the GWAS analysis concerning this trait was very limited due to the small number of individuals (332 fish) to detect even medium SNP effects. If a larger group was available, an SNP associated with disease resistance could be detected, as indeed happened with VNN disease in the same species in LG12 [24].

### 4.6. Genomic Selection and Comparison with Pedigree-Based Approach

Starting with the stress indicator, higher accuracy was noticed using GRM than the PRM matrix except for lysozyme levels, which showed equal findings; glucose levels had a higher accuracy when estimated using PRM. However, when a pairwise *t*-test was performed on accuracy between the two relationship matrices for glucose levels, the difference was not statistically significant (data not shown). Focusing on lysozyme levels, apart from the equal accuracy estimates which were detected, the highest correlation between EBVs and GEBVs was also found. On the other hand, cortisol levels showed higher prediction accuracy using GRM, but a lower correlation between EBVs and GEBVs was detected. Additionally, when the same square root of heritability ($\surd h_{PRM}^{²}$) was used in both models for accuracy for GEBVs and EBVs, higher estimations were noticed in all stress indicators, except for glucose levels.

For the body weight, the estimation accuracy was similar between the two methods (PRM and GRM) for the first two measurements, but for the later growth stages, the accuracy of prediction was slightly higher when using PRM compared to GRM. Based on the literature, the use of the GRM increases the accuracy of estimation; however, using this dataset, the findings did not confirm the pattern. When a pairwise *t*-test was performed on accuracy between the two relationship matrices for each measurement, the difference was not statistically significant. An explanation for this difference in accuracy could be the limited number of offspring and the use of only one generation. When the second approach was used (using the same square root of heritability ($\surd h_{PRM}^{²}$) in both models for accuracy estimation for GEBVs and EBVs), higher estimations were noticed in measurements of the body weight, but even then, the difference was not statistically significant.

### 4.7. Limitations and Further Research

In this study, there is some evidence of QTL affecting lysozyme and lactate levels in chr 17, 19, and 20. Lysozyme levels have been used as stress indicators for the Atlantic salmon and the rainbow trout [9,10,12], and increased levels have been noticed after stress exposure [58]. In physiology, the lactate levels rise when there is increased fish activity [59] and have been used as a stress indicator for the European seabass. After the stress experiment, an increase in lactate levels was identified [13,15]. However, whether lysozyme and lactate levels could be used as stress markers in a selective breeding program on their own or in combination between them as well as with other traits needs to be further investigated both genetically and physiologically.

Focusing on the limitations of the GWAS and genomic selection, the main points are the small number of fish (862) participating in the analysis and the structure of the data (i.e., split into two unequal batches). The use of the batch as the fixed effect was necessary because a significant difference between the average measurements of the trait was observed; moreover, there was population stratification between batch 10 and 13 (PCA analysis was performed, Supplementary File S2, Figure S12), and the farming period was different.

Apart from the limitations mentioned above, the consideration of increasing the sample size remains a limit as it is not easy to perform large-scale stress test experiments in the industry. Nevertheless, there are studies with smaller sample sizes that reported QTL affecting not only body weight but also cortisol and glucose levels, such as when Chatziplis et al. [17] used microsatellite markers with linkage analysis.

## 5. Conclusions

Five SNP were found statistically significant, three of which affecting stress indicators and body weight (into a subgroup of the population), and a putative SNP affecting growth performance, while no SNP associated with resistance to *Vibrio* was found. A moderate to high genomic heritability regarding stress indicators and body weight was estimated. The heritability of the stress indicators estimated using the GRM provided approximately similar estimates using the PRM, except for glucose levels. A high correlation between EBVS and GEBVs was detected for stress response and body weight. Accuracy of estimation using Genomic Selection (GS) was higher than the traditional pedigree approach for the body weight and stress indicators, except for the glucose levels.

## Figures and Tables

**Table 1:** Descriptive statistics for the traits.

	Batch		Batch 10 and 13			Batch 13		Batch 10	
Traits	Number of Observations Per Trait	Age (DPH *)	Number of Offspring	MEAN	SD	Mean	SDV	Mean	SD
Weight (g)	1	290–306	862	53.87	16.86	48.35	12.9	57.3	18.08
	2	318–334	858	65.26	20.85	60.35	16.37	68.28	22.68
	3	346–362	861	79.25	25.78	74.62	20.46	82.12	28.22
	4	362–378	861	93.02	32.78	86.9	24.28	96.8	36.59
Cortisol levels (ng mL^−1^)	1	318–334	859	339.43	79.77	300.54	68.09	363.57	76.96
	2	346–362	859	316.69	79.77	328.96	78.3	309.11	79.8
	3	362–378	860	313.33	83.95	339.47	78.39	297.21	83.27
Glucose levels (mmol L^−1^)	1	318–334	848	6.78	2.25	6.07	1.85	7.24	2.36
	2	346–362	854	7.02	2.1	6.65	1.93	7.25	2.18
	3	362–378	852	7.3	2.15	6.5	1.81	7.8	2.2
Lactate levels (mmol L^−1^)	1	318–334	861	6.29	3.39	5.73	2.13	6.64	3.94
	2	346–362	859	6.79	4.17	5.46	2.5	7.6	4.75
	3	362–378	858	7	4.69	4.93	2.23	8.27	5.31
Lysozyme levels (Kui^−1^)	1	318–334	817	568.2	287.45	408.73	168.23	671.94	301.22
	2	346–362	815	602.19	283.89	531.16	220.02	648.83	310.43
	3	362–378	814	619.79	317.26	568.2	217.93	653.56	364.32

* Days Post Hatching.

**Table 2:** Heritability (h^2^) and repeatability per trait per model for biochemical and homological factors. Standard errors are illustrated in the parentheses.

Stress Indicator	h^2^ (Using PRM *)	Repeatability (Using PRM *)	h^2^ (Using GRM **)
Cortisol levels	0.45 (0.02)	0.48 (0.02)	0.43 (0.06)
Glucose levels	0.31 (0.04)	0.32 (0.04)	0.52 (0.06)
Lactate levels	0.61 (0.05)	0.62 (0.05)	0.59 (0.06)
Lysozyme levels	0.63 (0.02)	0.64 (0.02)	0.75 (0.06)

* Pedigree relationship matrix. ** Genomic relationship matrix.

**Table 3:** Heritability (h^2^) and genetic/phenotypic correlation for body weight using pedigree relationship matrix (PRM). Heritability is on the diagonal in bold; genetic and phenotypic correlations are above the diagonal (in blue) and below (in green), respectively. Standard errors are illustrated in the parentheses.

	Weight 1	Weight 2	Weight 3	Weight 4
**Weight 1**	**0.75 (0.09)**	0.98 (0.01)	0.93 (0.03)	0.87 (0.04)
**Weight 2**	0.95 (0.05)	**0.70 (0.09)**	0.97 (0.01)	0.94 (0.02)
**Weight 3**	0.88 (0.01)	0.95 (0.00)	**0.55 (0.08)**	0.99 (0.00)
**Weight 4**	0.81 (0.01)	0.91 (0.01)	0.98 (0.01)	**0.54 (0.08)**

**Table 4:** Heritability (h^2^) and genetic/phenotypic correlation for body weight using genomic relationship matrix (GRM). Heritability is on the diagonal in bold; genetic and phenotypic correlations are above the diagonal (in blue) and below (in green), respectively. Standard errors are illustrated in the parentheses.

	Weight 1	Weight 2	Weight 3	Weight 4
**Weight 1**	**0.61 (0.06)**	0.97 (0.01)	0.90 (0.02)	0.83 (0.03)
**Weight 2**	0.95 (0.05)	**0.60 (0.06)**	0.97 (0.01)	0.93 (0.02)
**Weight 3**	0.88 (0.01)	0.95 (0.00)	**0.54 (0.06)**	0.99 (0.00)
**Weight 4**	0.81 (0.01)	0.91 (0.01)	0.98 (0.01)	**0.57 (0.06)**

**Table 5:** Significant SNPs in batch 10 and 13 from univariate and repeated GWAS.

Trait (Measurement)	Chr	SNP	Position (bp)	MAF	SNP Effect	SE	-log (*p*-Value)	PVE *** (%)
Lactate levels (Repeated measurements) *	19 (LG5)	AX-172290333	25,862,514	0.15	1.46	0.27	7.03	2.6
Lactate levels (3rd **)	17 (LG3)	AX-172304113	6167,189	0.075	2.47	0.47	6.63	2.5
Lactate levels (3rd **)	19 (LG5)	AX-172290333	25,862,514	0.15	1.84	0.35	6.69	2.5
Lysozyme levels (1st **)	20 (LG6)	AX-172274981	7284,104	0.2	99.18	19.87	6.12	2.2

* GWAS with repeated measurements. ** Univariate GWAS. *** Phenotypic variation explained.

**Table 6:** Significant SNPs in batch 10 from univariate GWAS.

Trait (Measurement)	Chr	SNP	Position (bp)	MAF	SNP Effect	SE	-Log (*p*-Value)	PVE * (%)
Weight (2nd)	16 (LG24)	AX-172310116	11,181,812	0.13	−11.01	2.12	6.51	2.4
Weight (2nd)	16 (LG24)	AX-172322981	11,223,301	0.28	−8.01	1.44	7.35	2.8
Weight (3rd)	16 (LG24)	AX-172310116	11,181,812	0.13	−15.16	2.81	6.98	2.6
Weight (3rd)	16 (LG24)	AX-172322981	11,223,301	0.28	−10.82	1.90	7.64	2.9
Weight (4th)	16 (LG24)	AX-172310116	11,181,812	0.13	−20.35	3.68	7.28	2.7
Weight (4th)	16 (LG24)	AX-172322981	11,223,301	0.28	−13.27	2.51	6.73	2.5
Lactate levels (3rd)	19 (LG5)	AX-172290333	25,862,514	0.21	2.33	0.46	6.15	2.3

* Phenotypic variation explained.

**Table 7:** Accuracy of estimation per trait per model. The standard error is illustrated in the parenthesis.

Trait	Accuracy with PRM ^†^ (EBVs)	Accuracy with GRM ^††^ (GEBVs)	Accuracy ^▪^ with GRM ^††^ (GEBVs) Using the $\surd h_{PRM}^{2}$
Cortisol levels	0.40 (0.04) *	0.51 (0.07) **	0.48 (0.06) **
Glucose levels	0.38 (0.03) *	0.28 (0.04) **	0.23 (0.04) **
Lactate levels	0.26 (0.05) *	0.31 (0.08) **	0.28 (0.08) **
Lysozyme levels	0.47 (0.07) *	0.47 (0.06) **	0.48 (0.06) **
Weight 1	0.54 (0.03)	0.60 (0.04)	0.62 (0.04)
Weight 2	0.53 (0.03)	0.56 (0.04)	0.62 (0.04)
Weight 3	0.43 (0.05)	0.41 (0.06)	0.55 (0.08)
Weight 4	0.42 (0.06)	0.40 (0.07)	0.54 (0.10)

* Using the repeated measurements, Equation (1). ** Using the corrected phenotypes, Equation (2). ^†^ Pedigree relationship matrix. ^††^ Genomic relationship matrix. ^▪^ Accuracy calculated using the square root of heritability estimated by BLUP (using PRM).

**Table 8:** Correlation between Estimated Breeding Values (EBVs) and Genomic Estimated Breeding Values (GEBVs) for the Body weight and stress indicators.

Trait	Correlation
Cortisol levels	0.79
Glucose levels	0.78
Lactate levels	0.78
Lysozyme levels	0.84
Weight 1	0.91
Weight 2	0.88
Weight 3	0.86
Weight 4	0.85

## Data Availability

The data presented in this study are available on request from the corresponding author. The data are not publicly available due to commercial restrictions.

## Supplementary Materials

The following are available online at https://www.mdpi.com/article/10.3390/ani12030277/s1, Supplementary File 1, Table S1 illustrates the number of the full sibs and half-sib families, Table S2 illustrates the number of offspring per family per batch, Table S3 illustrates the SNPs and average position per chromosome and Table S4 illustrates the list of the genes from closed regions to significant SNP. Supplementary File S2 includes the Manhattan plots for all the univariate GWAS for the body weight (Figure S1), the stress indicators (for each measurement) (Figures S2–S5), and the corrected phenotypes (Figures S6–S9) for batches 10 and 13, Manhattan plots for all the univariate GWAS for the body weight (Figure S10) and the lactate levels (Figure S11) for batch 10 and finally, PCA analysis of the studied population (Figure S12).
