# Supplementary material for: Genomic Selection and Genome-Wide Association Analysis for Stress Response, Disease Resistance and Body Weight in European Seabass
Source: Animals (Basel). 2022 Jan 23;12(3):277. doi: 10.3390/ani12030277 (PMC8833606; doi:10.3390/ani12030277)
Supplement: Supplementary file 1 [file animals-12-00277-s001.zip › S1_File.pdf]

Supplementary File S1

Table S1. Population Structure

Batch	Full Sibs	Half Sibs	Total Families
10	9 Families	53 Families	62
13	5 Families	15 Families	20
Total Families	14	68	82

Table S2. Number of offspring per batch

Batch	Offspring
10	From 2 to 10
13	From 15 to 17

Table S3. SNPs and average position per chromosome

Chromosome	Average	Number of SNPs
1 (LG10)	13,699.71	1,756
2 (LG11)	13,182.18	1,989
3 (LG12)	12,839.28	1,810
4 (LG13)	13,250.86	2,085
5 (LG14)	12,067.75	2,353
6 (LG15)	13,112.35	1,950
7 (LG16)	14,626.71	1,764
8 (LG17)	11,526.20	1,985
9 (LG18-21)	12,770.15	1,289
10 (LG19)	12,601.07	1,842
11 (LG1A)	12,570.89	2,309
12 (LG1B)	12,550.01	1,433
13 (LG2)	12,399.05	2,112
14 (LG20)	14,668.08	1,936
15 (LG22-25)	13,745.70	1,924
16 (LG24)	9,876.46	1,409
17 (LG3)	13,233.85	1,014
18 (LG4)	13,506.98	2,042
19 (LG5)	14,571.99	2,238
20 (LG6)	13,417.28	2,100
21 (LG7)	13,329.40	2,136
22 (LG8)	12,790.06	1,804
23 (LG9)	12,278.40	1,821
24 (LGx)	11,180.64	1,590
25 (UN)	17,796.25	5,445

Table S4. List of the genes from closed regions to significant SNP

SNP	Affected phenotype	Linked Genes to the sequence of SNPs
AX-172304113	Lactate levels	*gpha2/grycoprotein hormone alpha 2*<br>*gphb5*
AX-172290333	Lactate levels	*mpped2/metallophosphoesterase domain containing 2*
AX-172274981	Lysozyme levels	*itpr2/ inositol 1,4,5-triphosphote receptor, type 2*<br>*sspn*
AX-172310116	Weight	*cdadc1/cytidine and CMP deaminase domain containing 1*<br>*rcbtb1/regulator of chromosome condensation (rcc1) & btb (poz) domain containing protein 1*
AX-172322981	Weight	fgf14/-<br>*rcbtb1/regulator of chromosome condensation (rcc1) & btb (poz) domain containing protein 1*

Table S5. Descriptive statistics per tank

Tank	W1	W2	W3	W4	Cortisol	Glucose	Lactate	Lysozyme
1	71.0	85.3	96.9	113.0	250.7	6.8	6.3	767.4
2	59.7	75.8	81.4	95.3	323.1	6.3	5.8	717.6
3	43.4	46.9	51.9	54.5	346.8	7.4	5.9	1246.2
4	59.4	65.2	70.3	77.2	357.4	6.0	4.5	308.5
5	62.8	65.9	69.4	72.8	367.5	6.6	8.1	704.1
6	53.1	67.2	81.1	91.9	365.0	9.1	8.3	883.9
7	50.3	61.4	73.2	89.8	271.0	6.4	5.9	931.1
8	58.5	73.9	92.9	109.5	279.1	7.2	14.8	673.8
9	56.9	71.4	86.2	109.3	324.3	9.3	7.7	952.1
10	62.1	75.8	88.1	107.3	351.5	6.6	5.7	243.3
11	45.6	48.5	60.0	69.1	282.1	5.0	6.3	1095.5
12	70.9	83.4	92.3	109.0	285.6	7.0	4.9	1014.7
13	61.3	71.5	82.0	94.4	289.6	6.4	6.1	266.3
14	52.7	63.3	81.0	99.7	352.1	7.0	8.4	838.3
15	47.0	57.7	72.8	87.3	294.2	9.8	4.3	901.9
16	49.9	66.2	88.1	108.5	360.4	4.7	5.0	1034.8
17	65.7	76.7	86.7	100.2	361.8	6.5	7.5	361.8
18	63.8	76.4	86.8	100.4	358.4	7.2	8.1	516.8
19	76.0	95.1	113.9	145.2	251.8	7.0	11.3	497.9
20	74.0	79.1	101.3	119.1	334.3	7.7	10.9	463.3
21	63.7	69.8	73.3	77.8	388.3	6.4	11.1	815.8
22	48.5	58.0	73.8	87.6	303.9	6.9	6.4	357.6
23	79.1	90.5	104.2	127.0	281.0	6.0	6.7	415.8
24	45.8	53.5	62.9	71.7	332.9	6.9	5.4	378.5
25	83.5	98.5	120.4	143.6	261.8	8.3	5.5	1011.5
26	52.4	60.3	70.1	76.7	353.8	6.6	4.7	667.0

27	45.1	53.1	68.4	83.6	417.5	6.5	6.0	361.4
28	58.9	74.7	87.7	107.7	411.2	7.1	8.7	985.2
29	67.6	78.6	91.8	104.6		7.4	7.6	533.0
30	66.8	81.1	95.8	114.4	300.6	7.2	4.5	338.6
31	47.3	58.8	78.0	96.2	313.0	8.1	10.6	196.9
32	72.1	87.7	104.1	125.8	359.1	9.6	9.0	483.9
33	59.8	77.7	101.9	122.5	343.4	8.3	7.9	802.0
34	27.3	33.8	44.8	54.8	373.0	6.5	3.7	473.1
35	34.7	41.7	56.4	71.2	430.1	7.3	11.1	504.3
36	48.8	56.8	72.0	85.8	280.1	8.9	8.5	524.3
37	39.7	47.9	57.6	64.7	372.3	7.1	4.1	1109.6
38	41.5	50.8	62.5	70.0	336.9	7.3	6.4	327.9
39	48.9	59.7	74.2	86.8	323.7	9.7	15.7	774.0
40	57.6	69.5	87.2	107.6	258.5	11.6	10.8	1076.5
41	49.3	55.6	79.0	86.8	407.0	7.0	7.6	554.7
42	56.4	68.8	85.6	101.7	310.0	5.4	4.0	439.3
43	51.8	58.6	69.0	79.3	339.6	6.9	9.4	1028.0
44	79.9	102.6	122.3	148.9	259.9	9.2	6.1	618.9
45	66.2	83.3	99.9	115.8	294.2	8.9	8.6	335.3
46	25.5	31.4	40.8	49.1		9.0	2.5	461.0
47	45.6	55.6	72.3	86.8	347.2	8.4	6.5	598.2
48	51.0	60.4	82.5	99.8	390.9	8.6	4.2	595.1
49	57.6	73.7	89.6	110.0	215.7	7.6	12.9	696.2
50	51.0	60.5	75.7	90.1	251.4	6.3	10.1	605.3
51	78.0	91.2	108.6	122.0	355.0	6.5	7.2	389.2
52	64.5	71.6	91.2	106.7	294.1	7.8	9.5	1098.5
53	70.6	76.9	84.7	94.0	294.2	8.7	10.7	573.7
54	31.8	40.1	52.5	64.7	331.7	7.1	7.4	727.7
55	80.0	95.2	109.7	128.6	303.4	6.3	13.2	573.3
56	69.7	80.9	91.7	106.5	315.6	7.7	5.3	842.2
57	57.9	65.6	78.5	91.9	230.2	7.1	5.3	669.6
58	46.1	52.1	61.3	68.5	329.1	8.3	7.9	794.5
59	54.3	62.1	70.4	74.2	290.2	5.9	7.7	1057.3
60	47.4	61.9	75.4	88.7	324.6	7.4	7.5	677.7
61	42.8	52.9	66.2	77.7	307.0	7.0	6.9	638.1
62	46.6	58.6	73.1	85.5	300.4	6.7	5.1	547.2
63	54.0	66.2	81.9	95.4	270.6	5.8	6.8	728.3
64	47.1	59.0	72.9	85.4	288.3	5.9	6.6	458.8
65	46.3	55.5	69.4	80.6	320.5	6.8	6.6	641.1
66	46.9	53.3	70.7	81.8	342.1	6.1	3.9	631.2
67	49.2	62.5	77.4	91.0	341.1	5.4	4.9	456.4
68	51.6	64.0	78.5	91.1	329.0	6.5	4.4	464.1
69	47.8	59.4	74.6	87.5	309.3	4.9	4.7	451.7
70	41.6	48.0	64.0	75.0	321.2	6.5	5.6	517.6

71	39.7	49.5	60.1	69.9	296.7	6.6	6.2	541.8
72	48.2	61.2	77.9	87.9	334.0	5.7	4.9	416.9
73	46.0	57.6	69.9	81.9	319.7	7.1	4.7	494.9
74	51.6	66.3	83.2	96.2	358.0	5.9	5.6	381.8
75	55.6	69.0	85.1	99.2	354.4	7.3	5.8	445.9
76	54.6	69.1	84.3	98.9	305.3	6.9	4.9	419.6
77	46.5	54.1	65.7	76.1	316.0	7.3	5.8	507.4
78	50.5	61.6	76.7	89.2	384.7	6.9	5.0	495.4
79	51.6	63.6	77.8	90.0	347.9	6.6	4.6	289.0
80	70.6	86.9	84.7	88.0				
81	48.4	56.9	51.9	54.5				
82	31.8	41.1	52.5	64.7				
