# Supplementary material for: Genomic Selection and Genome-Wide Association Analysis for Stress Response, Disease Resistance and Body Weight in European Seabass
Source: Animals (Basel). 2022 Jan 23;12(3):277. doi: 10.3390/ani12030277 (PMC8833606; doi:10.3390/ani12030277)
Supplement: Supplementary file 1 [file animals-12-00277-s001.zip › S2_File.pdf]

Supplementary File S2

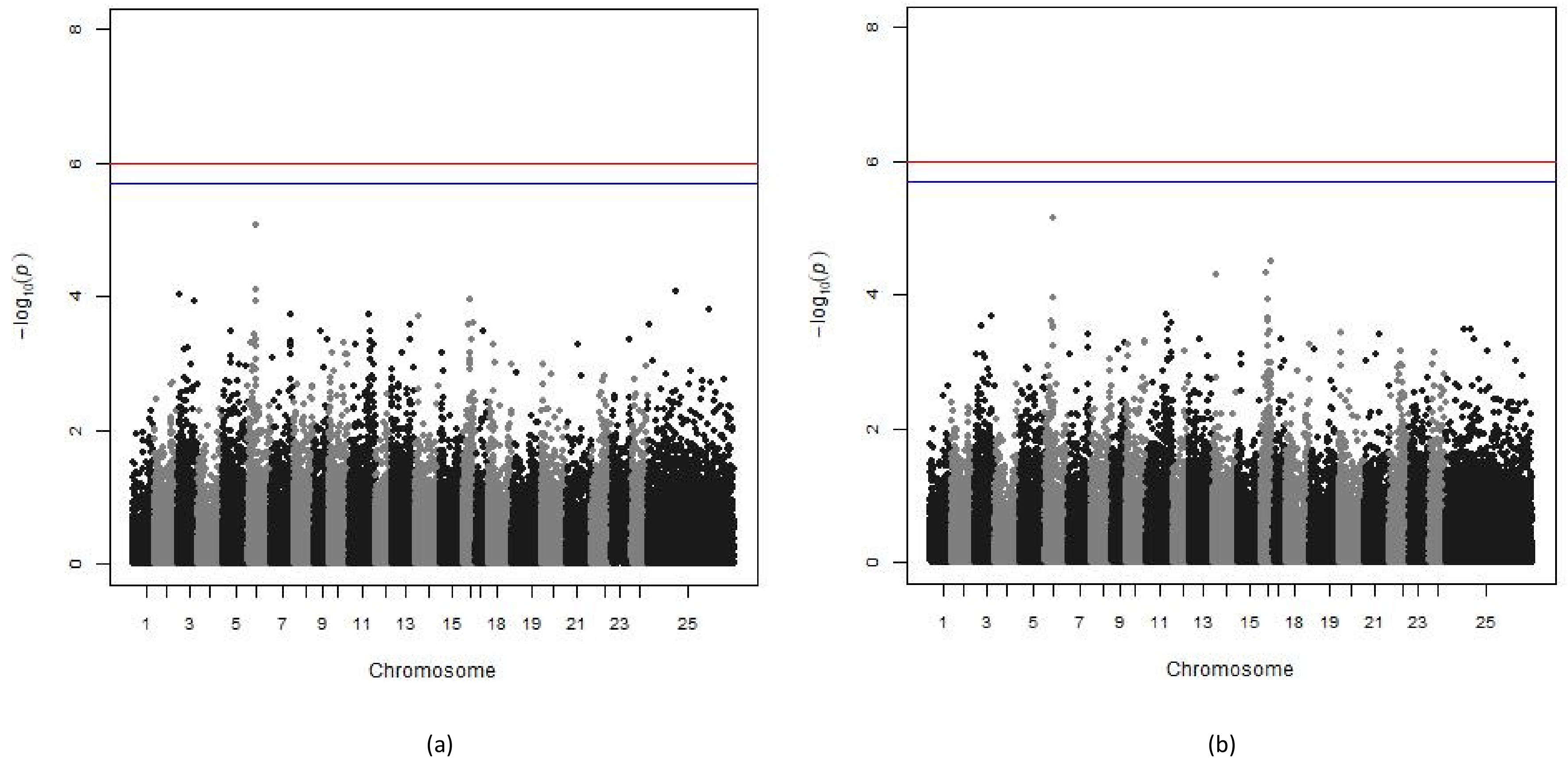

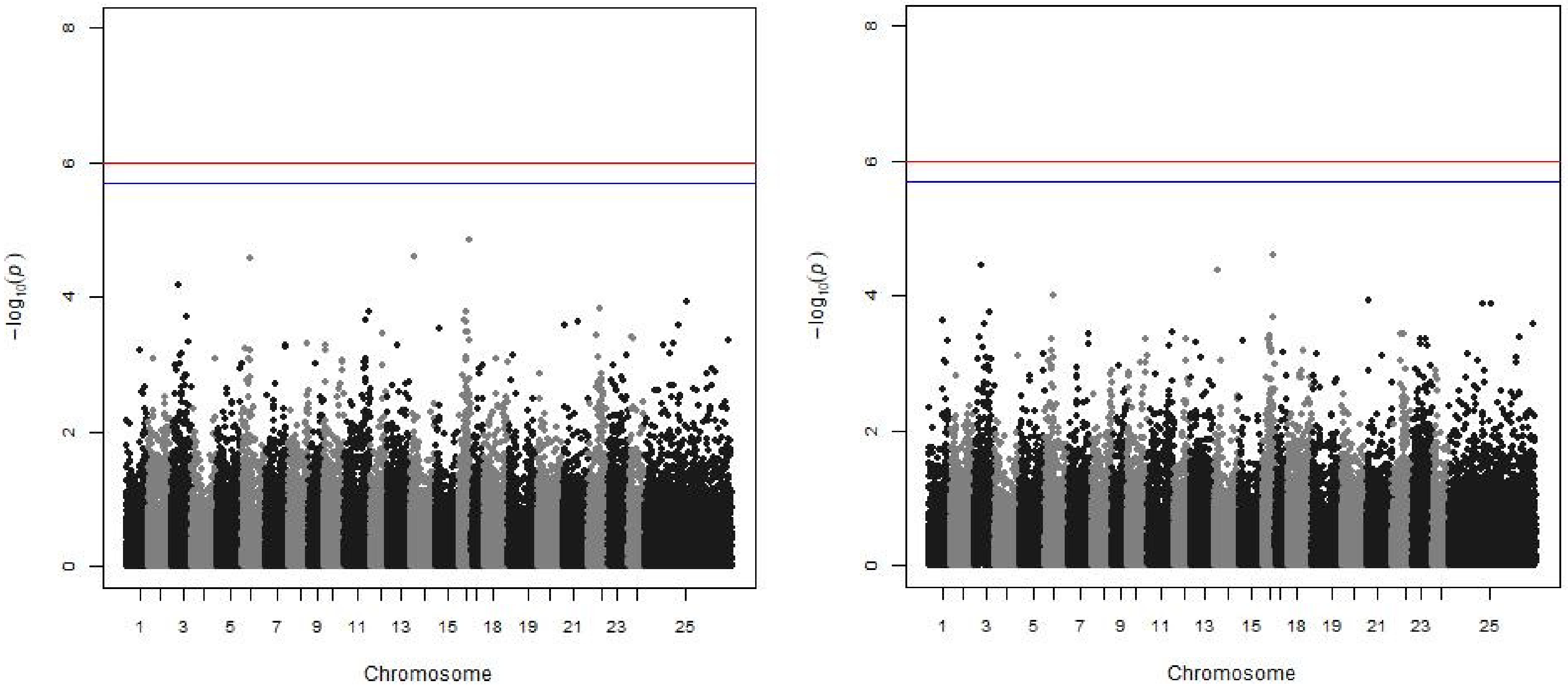

(c) (d)

Figure S1. Manhattan plots for body weight for batch 10 and 13. (a) illustrates the first measurement of the body weight, (b) shows the second measurement of the body weight, (c) illustrates the third measurement of the body weight and (d) shows the last measurement of the body weight. The red (initial value 0.05) and blue lines (initial value 0.1) illustrate the threshold, after Bonferroni correction.

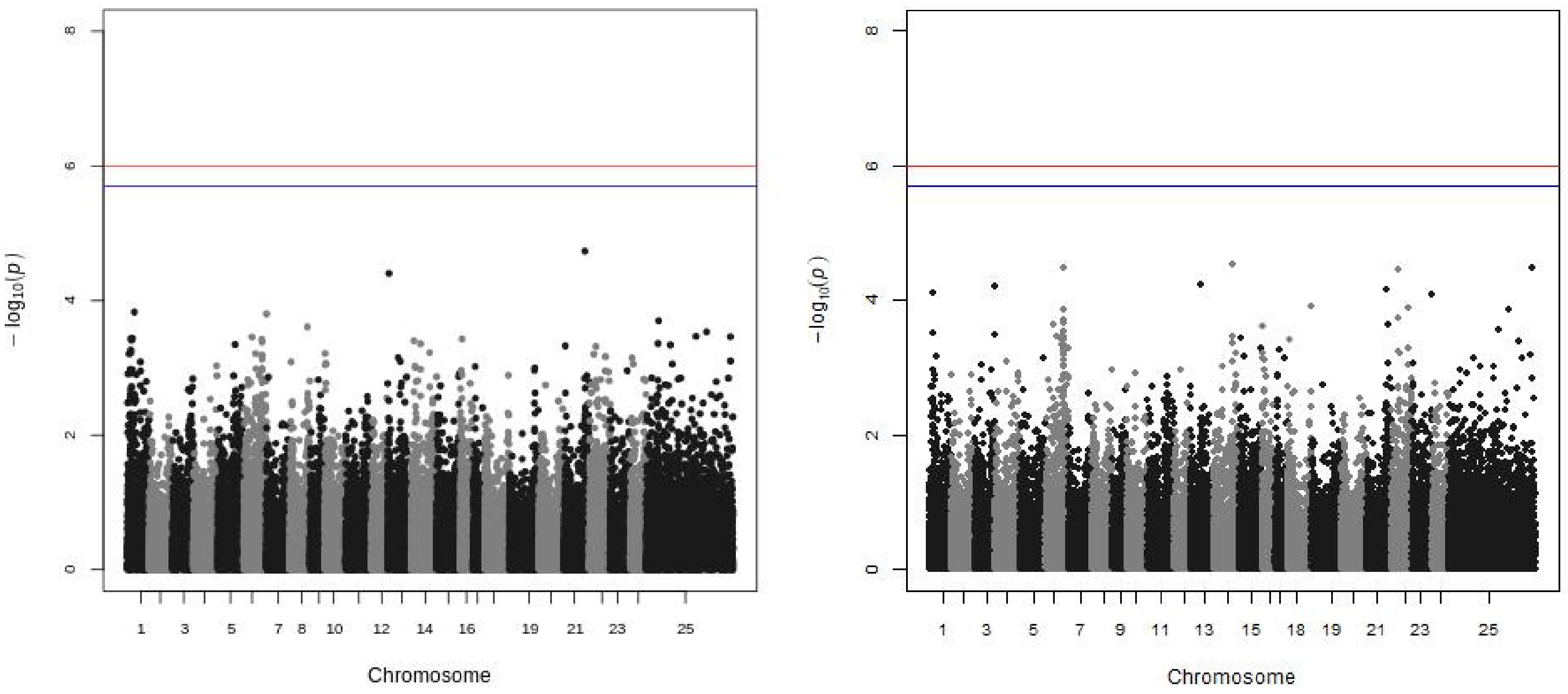

8
6
4
2
0
−log10(p)
1 3 5 7 8 10 12 14 16 19 21 23 25
Chromosome
8
6
4
2
0
−log10(p)
1 3 5 7 9 11 13 15 18 19 21 23 25
Chromosome

(a) (b)

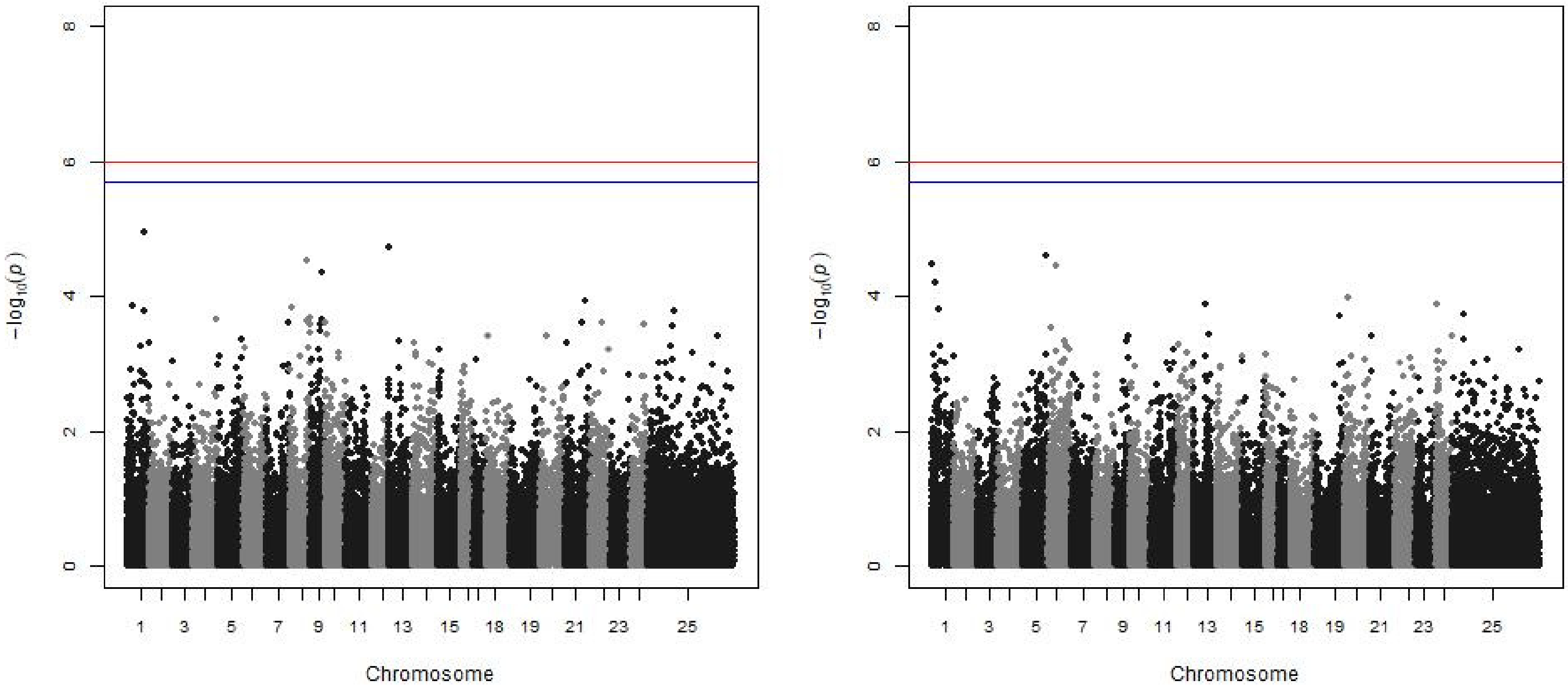

(c) (d)

Figure S2. Manhattan plots for cortisol levels for batch 10 and 13. (a) illustrates the corrected phenotypes, (b) shows the first measurement of the cortisol levels, (c) illustrates the second measurement and (d) shows the last measurement. The red (initial value 0.05) and blue lines (initial value 0.1) illustrate the threshold, after Bonferroni correction.

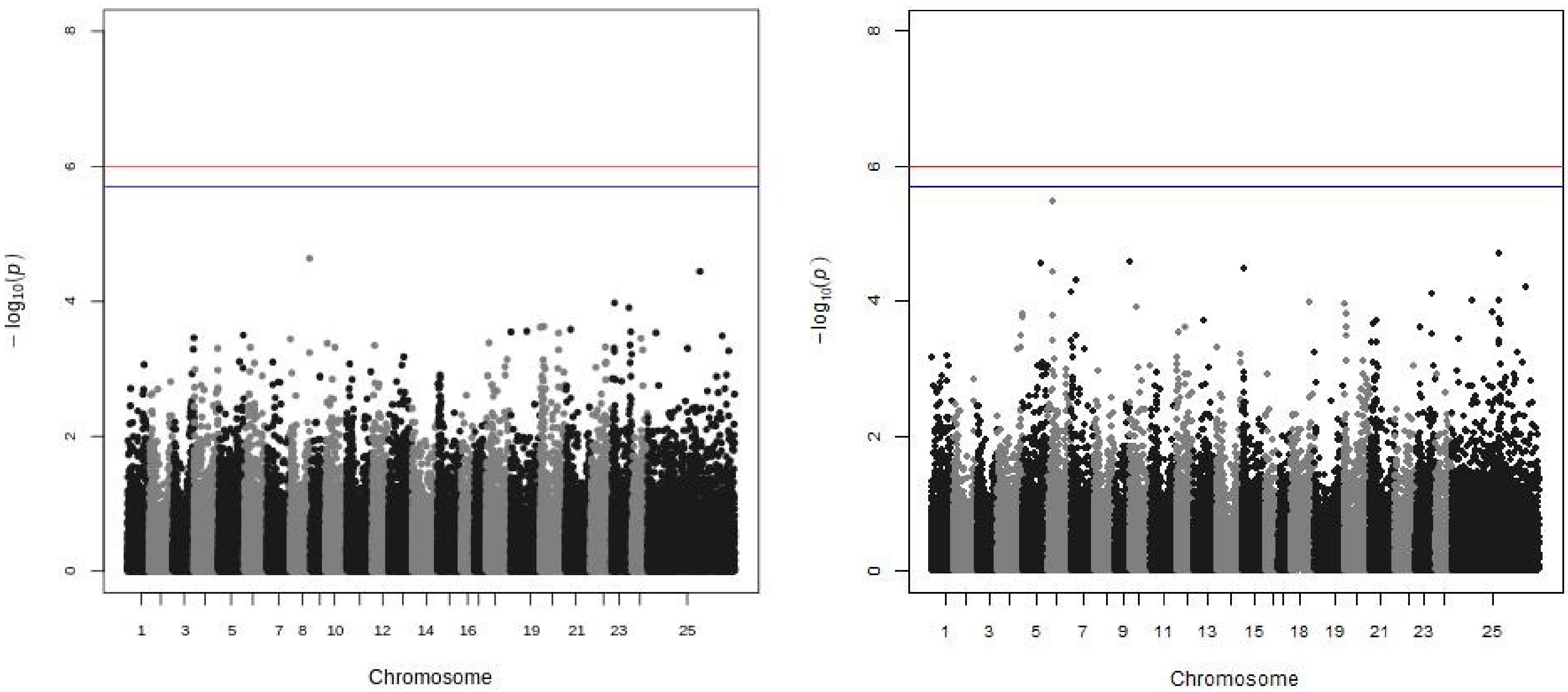

(a)

(b)

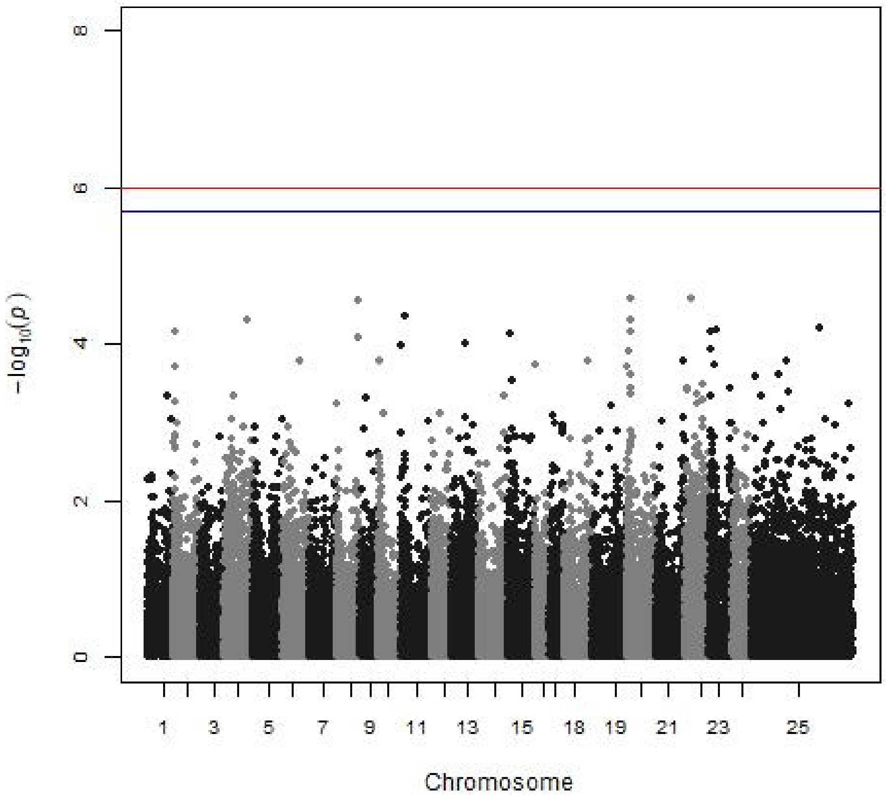

(c)

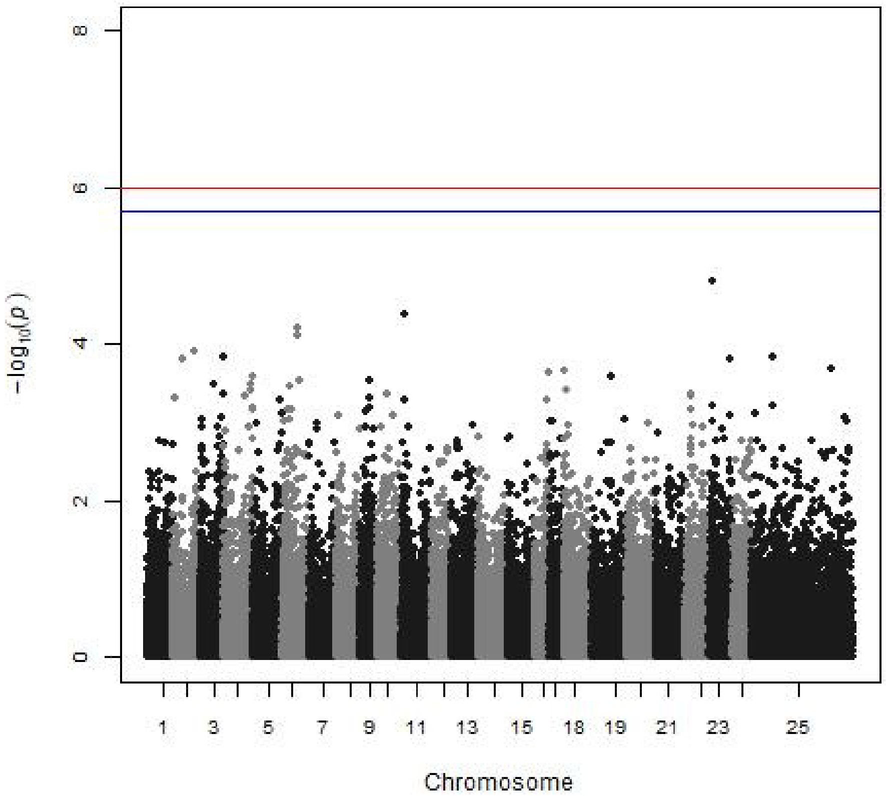

(d)

Figure S3. Manhattan plots for glucose levels for batch 10 and 13. (a) illustrates the corrected phenotypes, (b) shows the first measurement of the glucose levels, (c) illustrates the second measurement and (d) shows the last measurement. The red (initial value 0.05) and blue lines (initial value 0.1) illustrate the threshold, after Bonferroni correction.

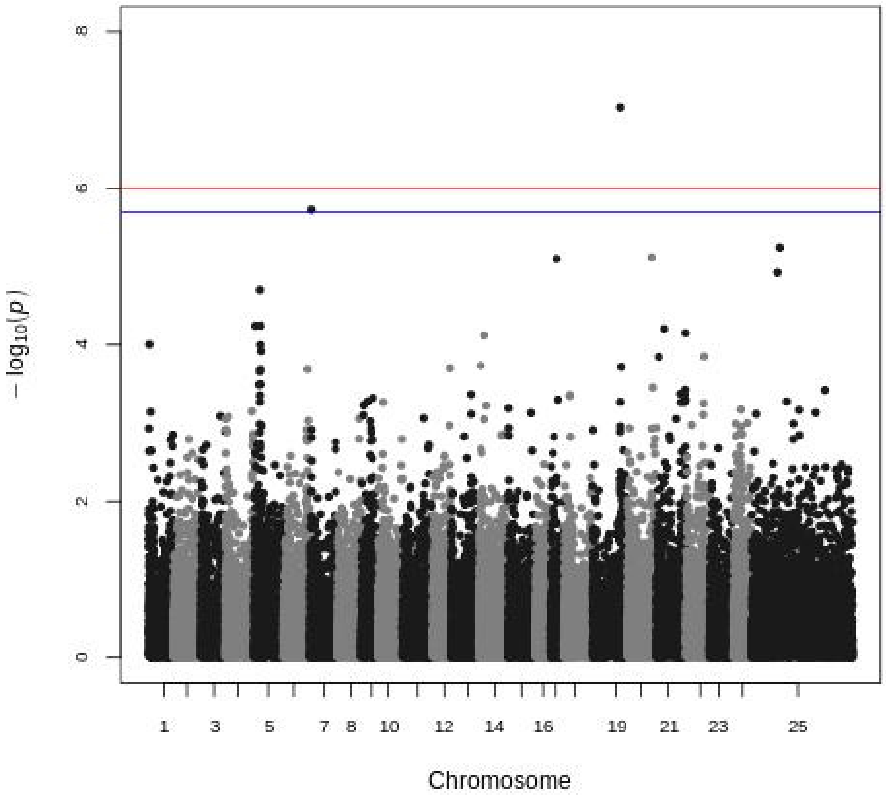
−log10(p)
0
2
4
6
8
1
3
5
7
8
10
12
14
16
19
21
23
25
Chromosome

(a)

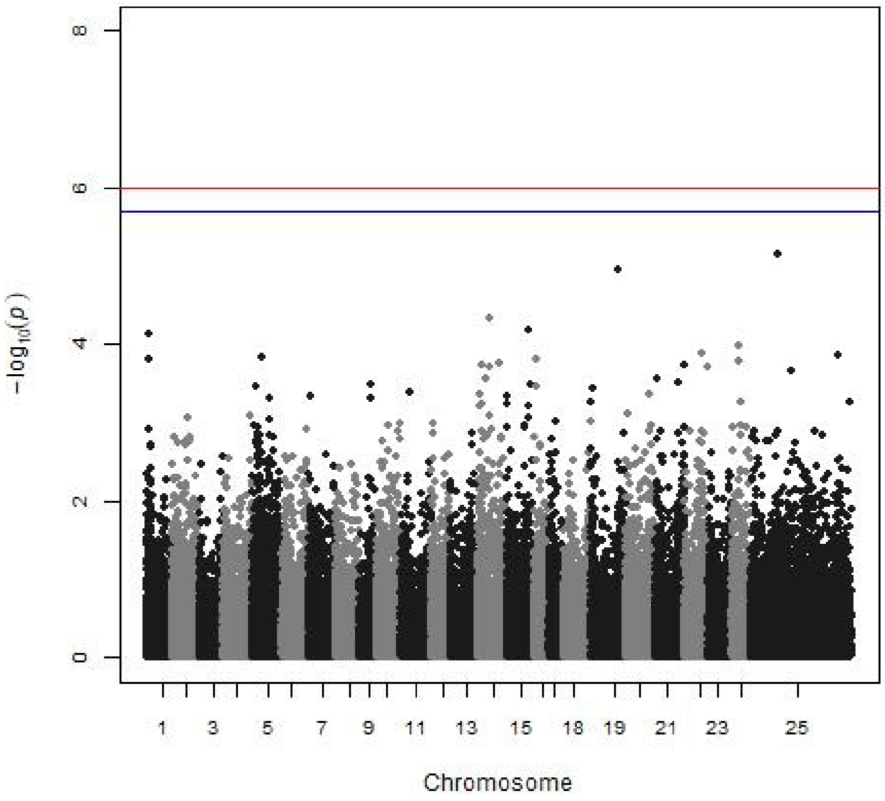
−log10(p)
0
2
4
6
8
1
3
5
7
9
11
13
15
18
19
21
23
25
Chromosome

(b)

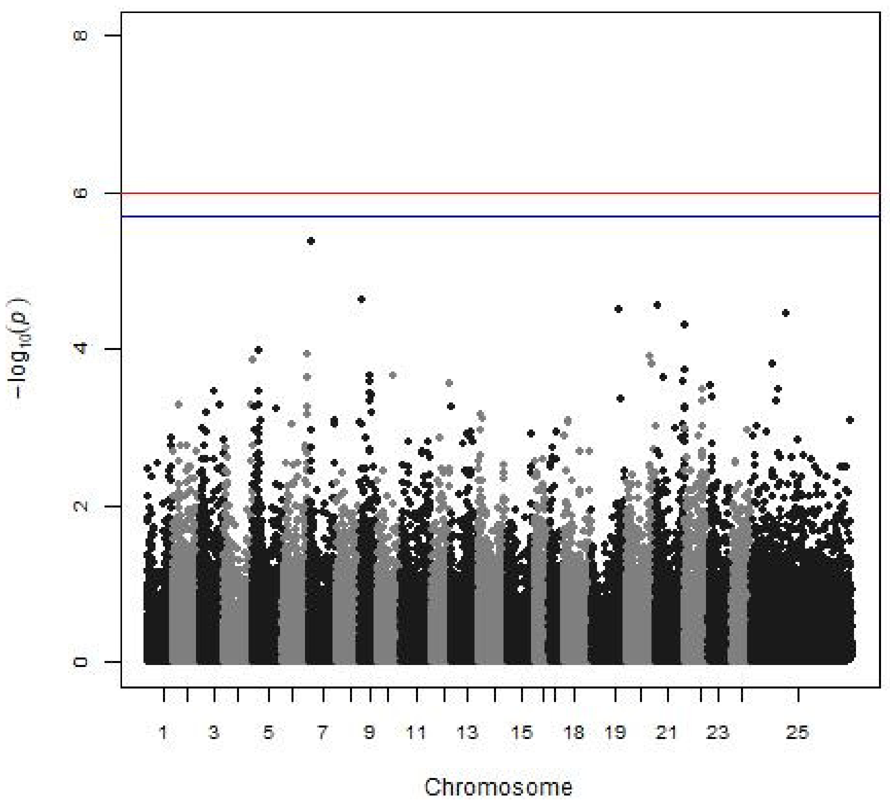

(c)

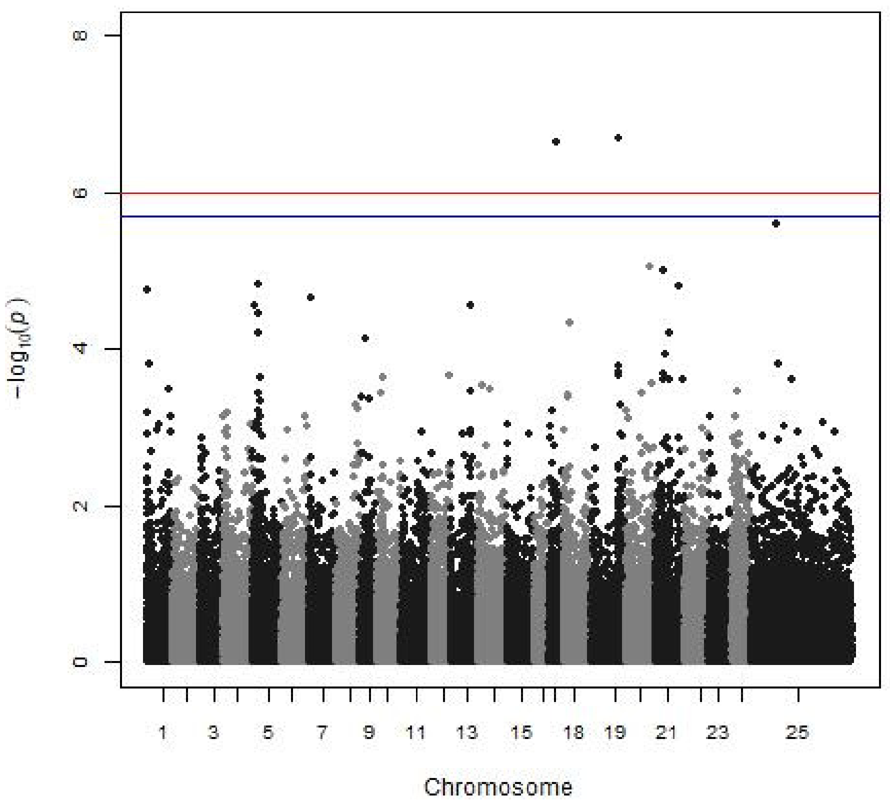

(d)

Figure S4. Manhattan plots for lactate levels for batch 10 and 13. (a) illustrates the corrected phenotypes, (b) shows the first measurement of the lactate levels, (c) illustrates the second measurement and (d) shows the last measurement. The red (initial value 0.05) and blue lines (initial value 0.1) illustrate the threshold, after Bonferroni correction.

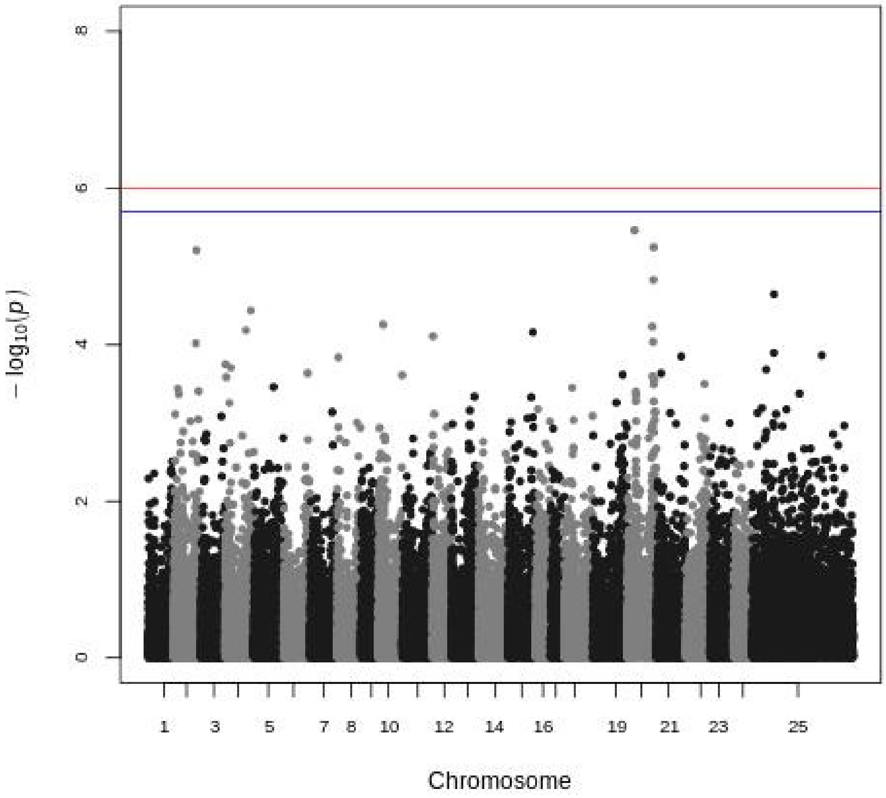

−log10(p)
0
2
4
6
8
1 3 5 7 8 10 12 14 16 19 21 23 25
Chromosome

(a)

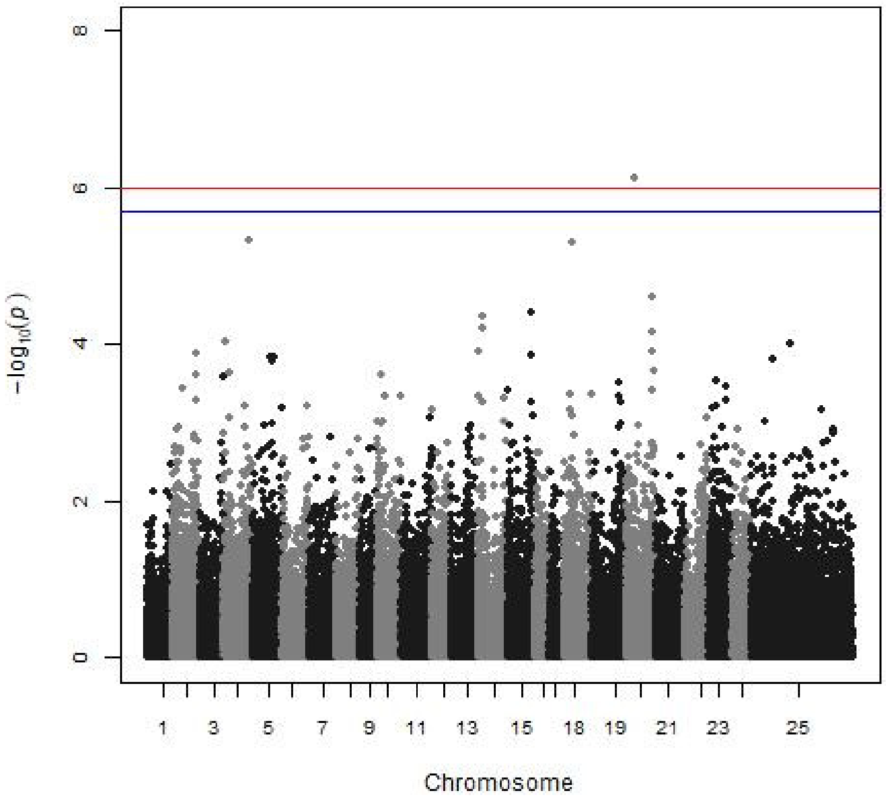

−log10(p)
0
2
4
6
8
1 3 5 7 9 11 13 15 18 19 21 23 25
Chromosome

(b)

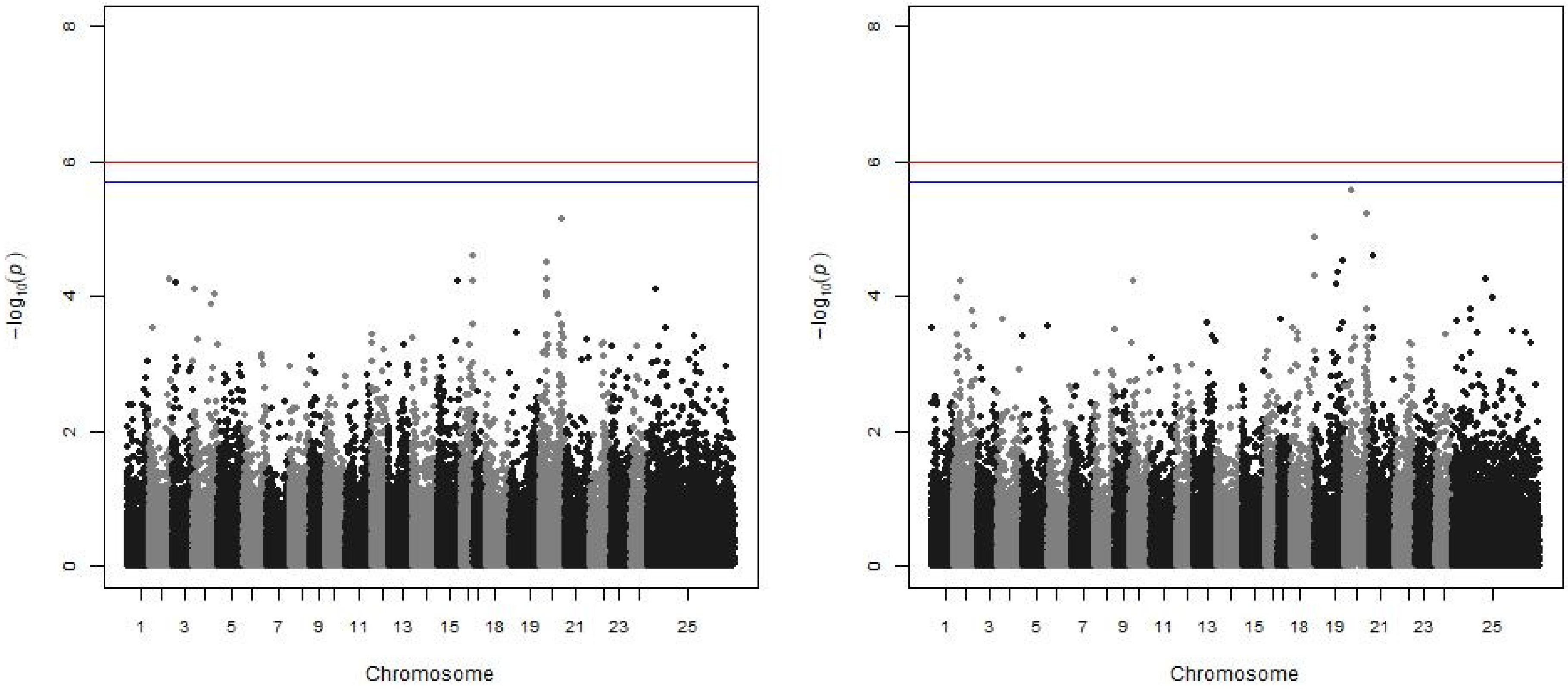

(c)

(d)

Figure S5. Manhattan plots for lysozyme levels for batch 10 and 13. (a) illustrates the corrected phenotypes, (b) shows the first measurement of the lysozyme levels, (c) illustrates the second measurement and (d) shows the last measurement. The red (initial value 0.05) and blue lines (initial value 0.1) illustrate the threshold, after Bonferroni correction.

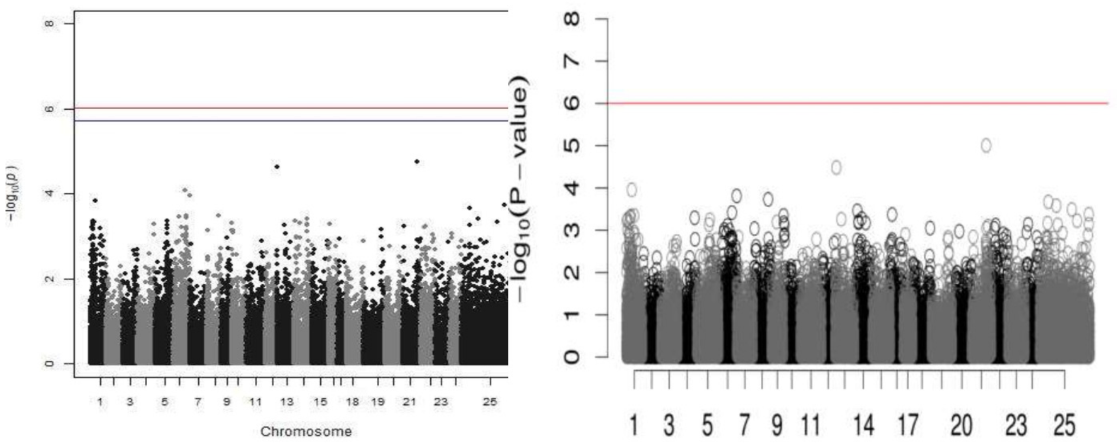

(a) (b)

Figure S6. Manhattan plots for cortisol levels for batch 10 and 13. (a) illustrates the results for the corrected phenotypes (b) shows the results for the repeated measurements.

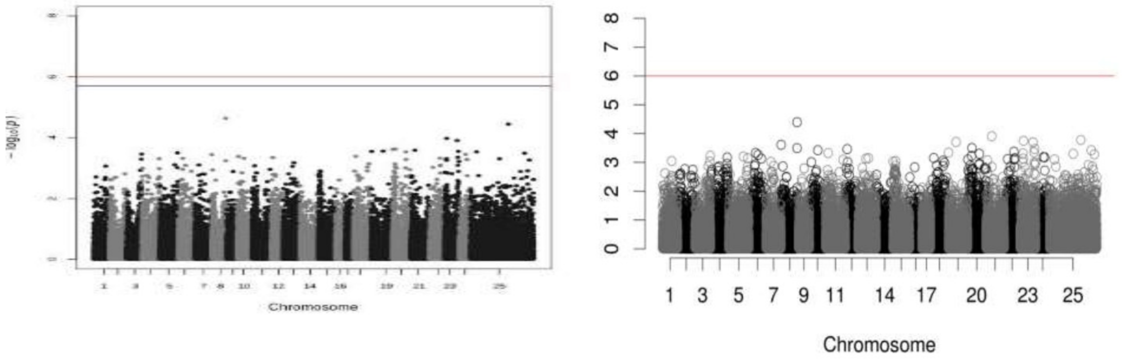

(a) (b)

Figure S7. Manhattan plots for glucose levels for batch 10 and 13. (a) illustrates the results for the corrected phenotypes (b) shows the results for the repeated measurements.

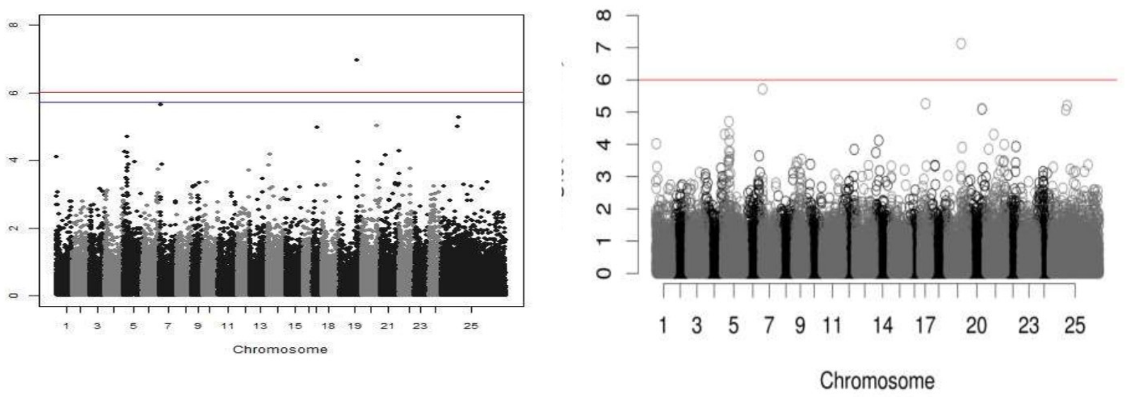

(a) (b)

Figure S8. Manhattan plots for lactate levels for batch 10 and 13. (a) illustrates the results for the corrected phenotypes (b) shows the results for the repeated measurements.

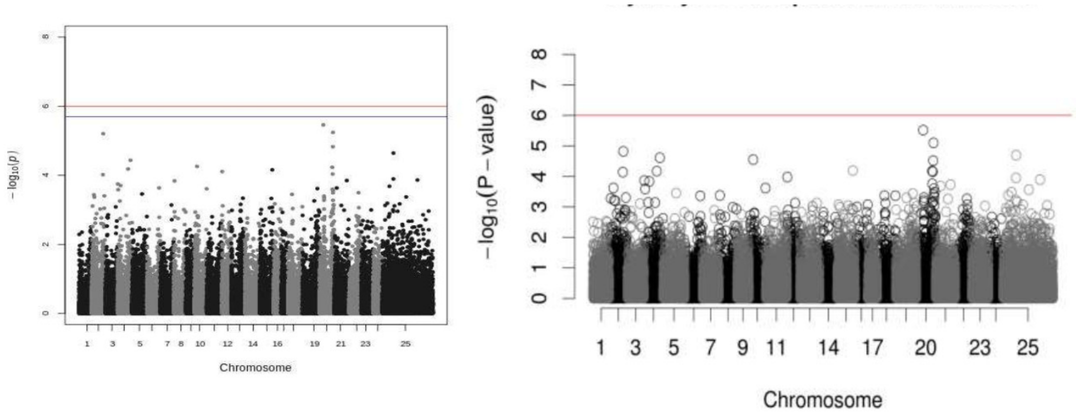

(a) (b)

Figure S9. Manhattan plots for lysozyme levels for batch 10 and 13. (a) illustrates the results for the corrected phenotypes (b) shows the results for the repeated measurements.

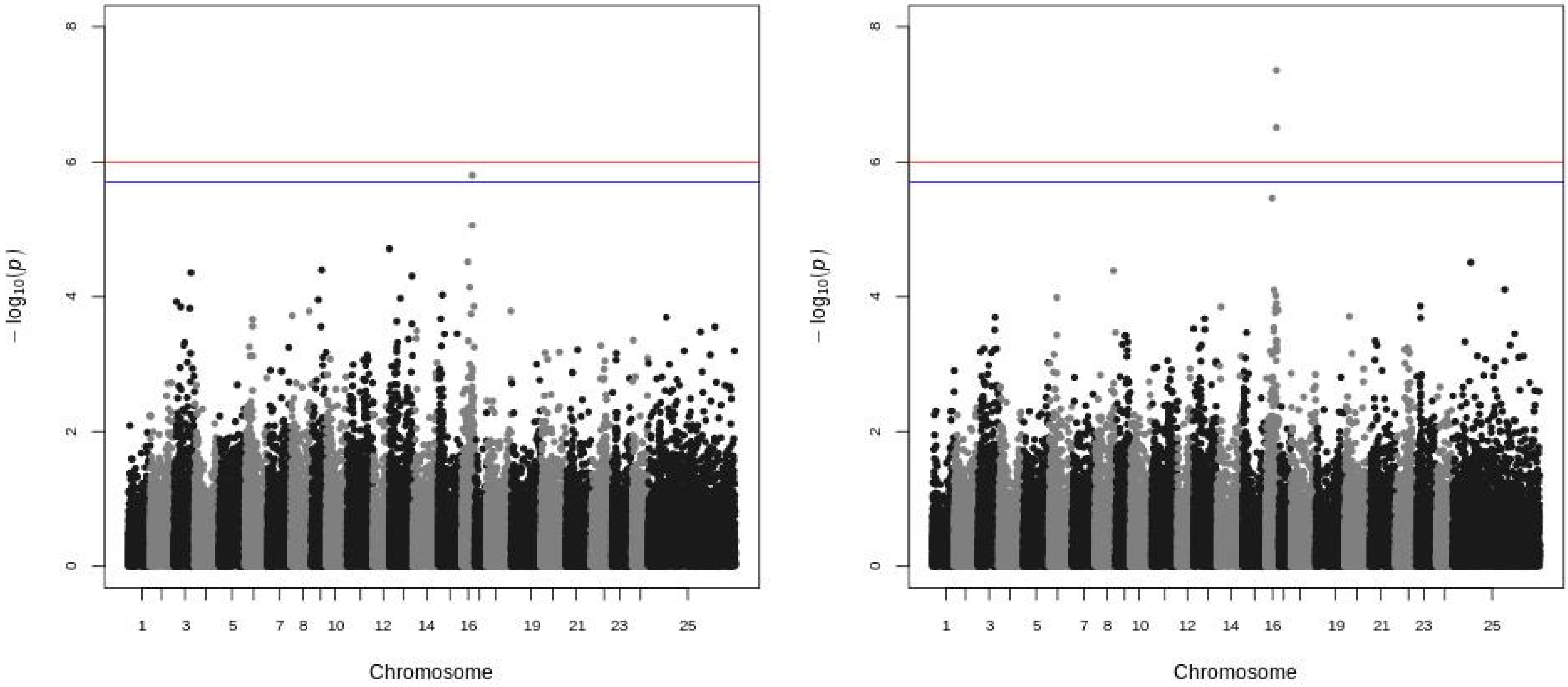

(a)

(b)

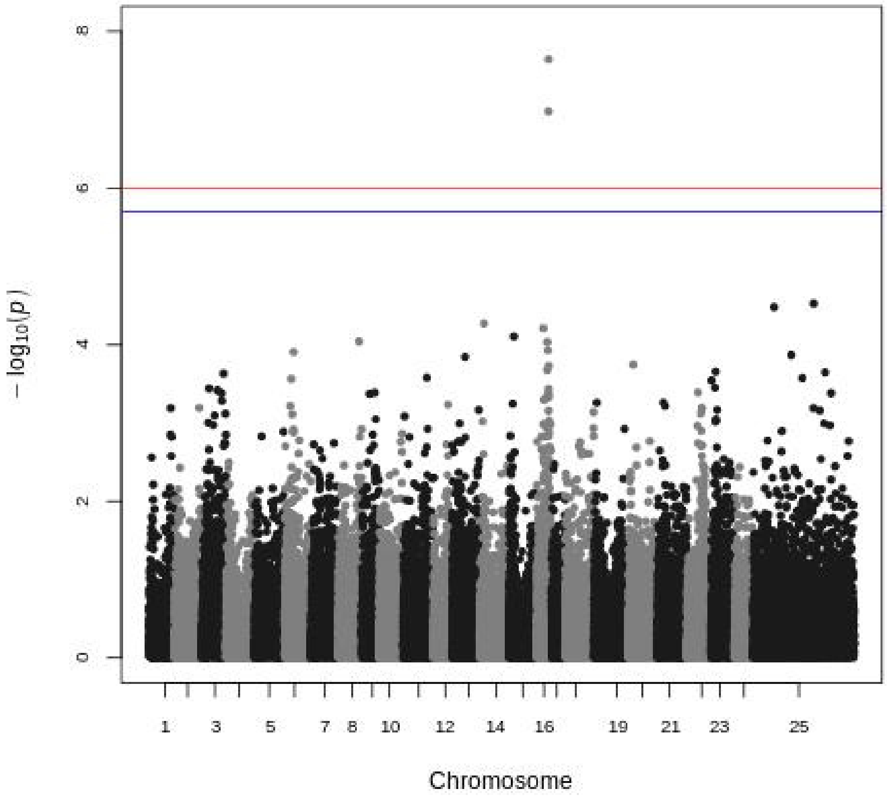

(c)

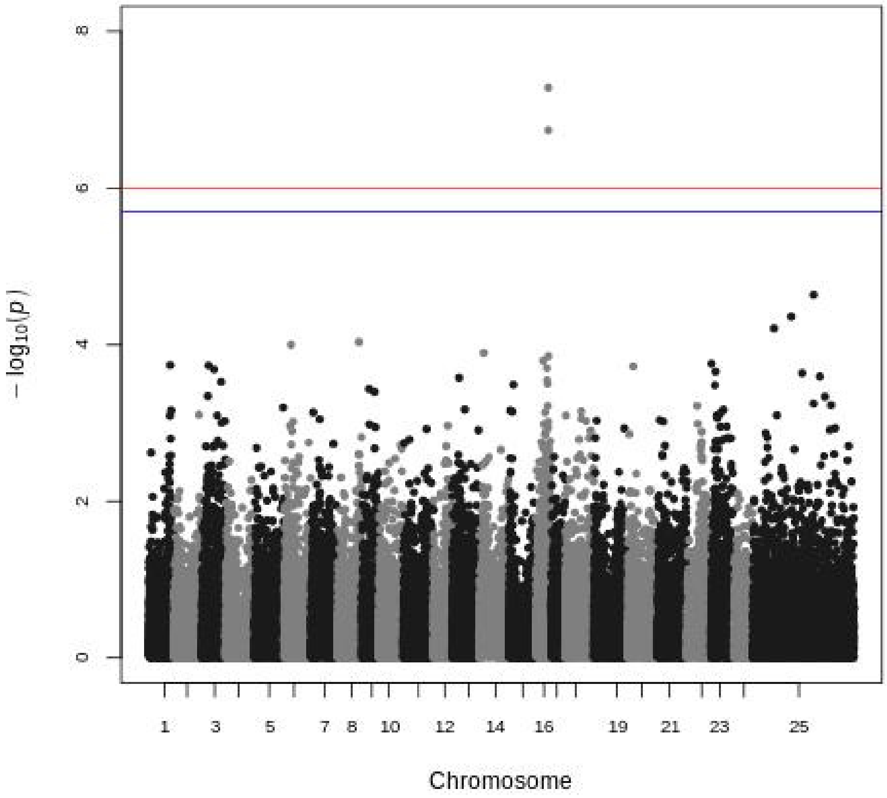

(d)

Figure S10. Manhattan plots for body weight for batch 10. (a) illustrates the first measurement of the body weight, (b) shows the second measurement of the body weight, (c) illustrates the third measurement of the body weight and (d) shows the last measurement of the body weight. The red (initial value 0.05) and blue line (initial value 0.1) illustrate the threshold, after Bonferroni correction.

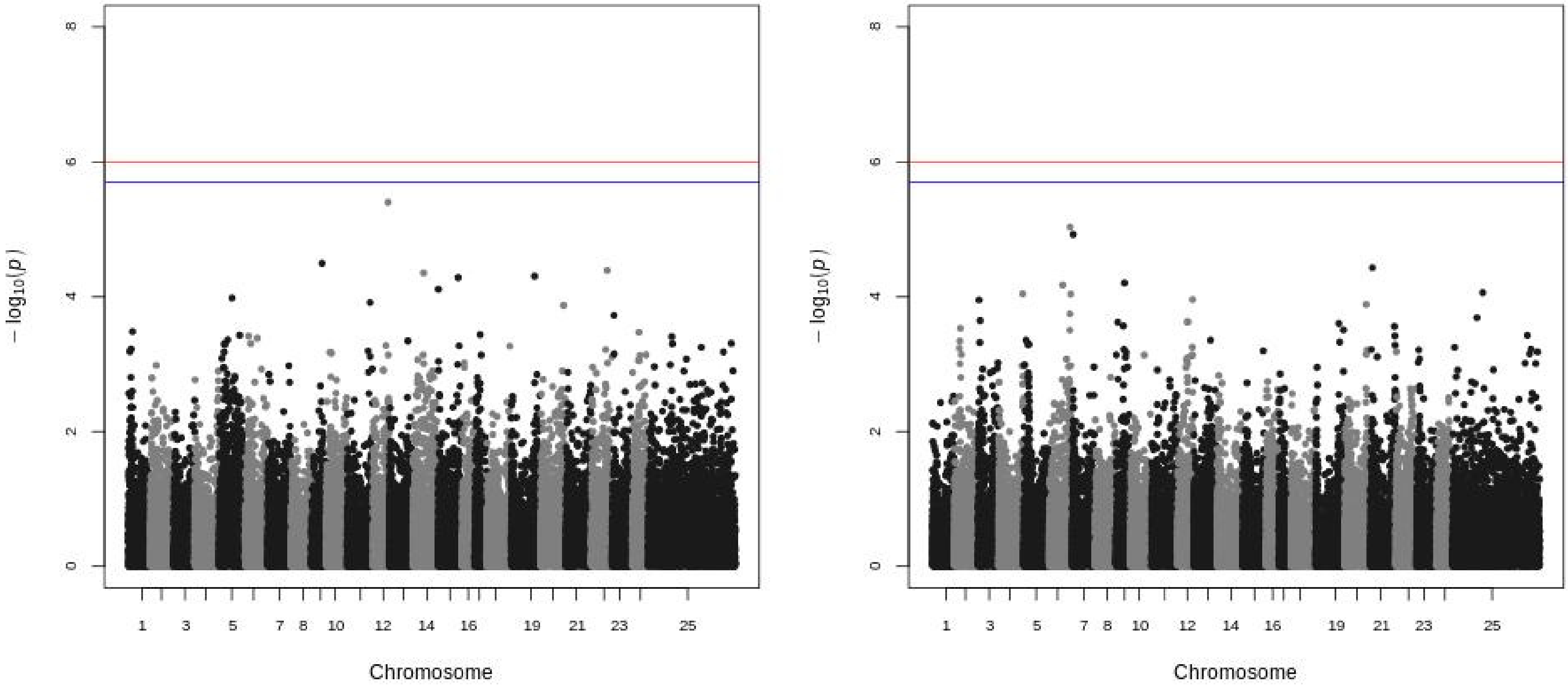

(a)

(b)

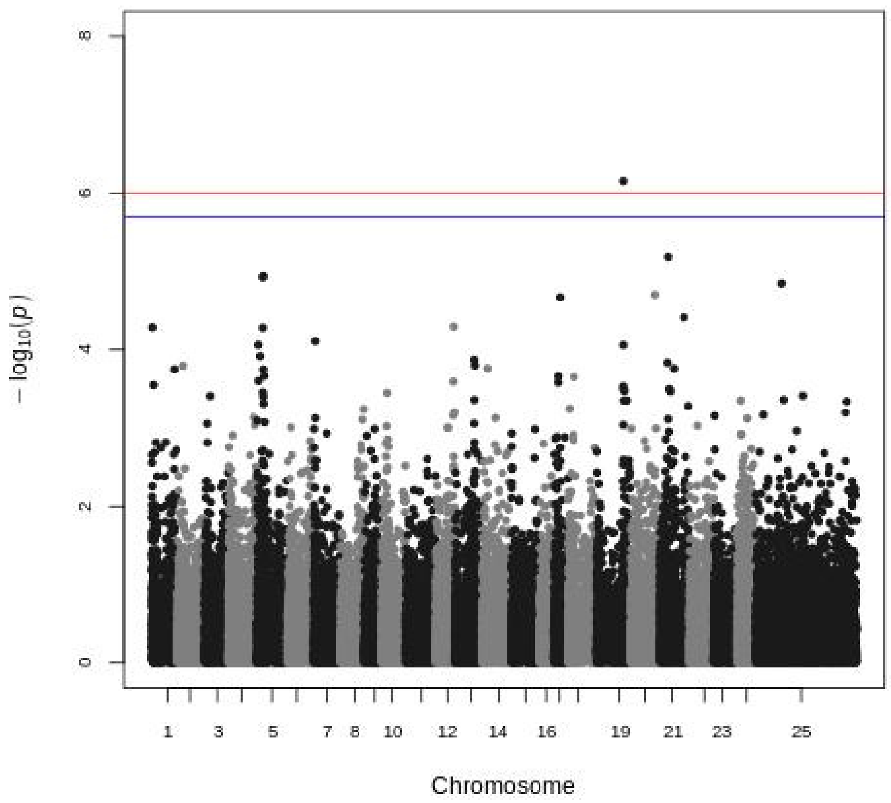

(c)

Figure S11. Manhattan plots for lactate levels for batch 10. (a) shows the first measurement of the lactate levels, (b) illustrates the second measurement and (c) shows the last measurement. The red (initial value 0.05) and blue lines (initial value 0.1) illustrate the threshold, after Bonferroni correction.

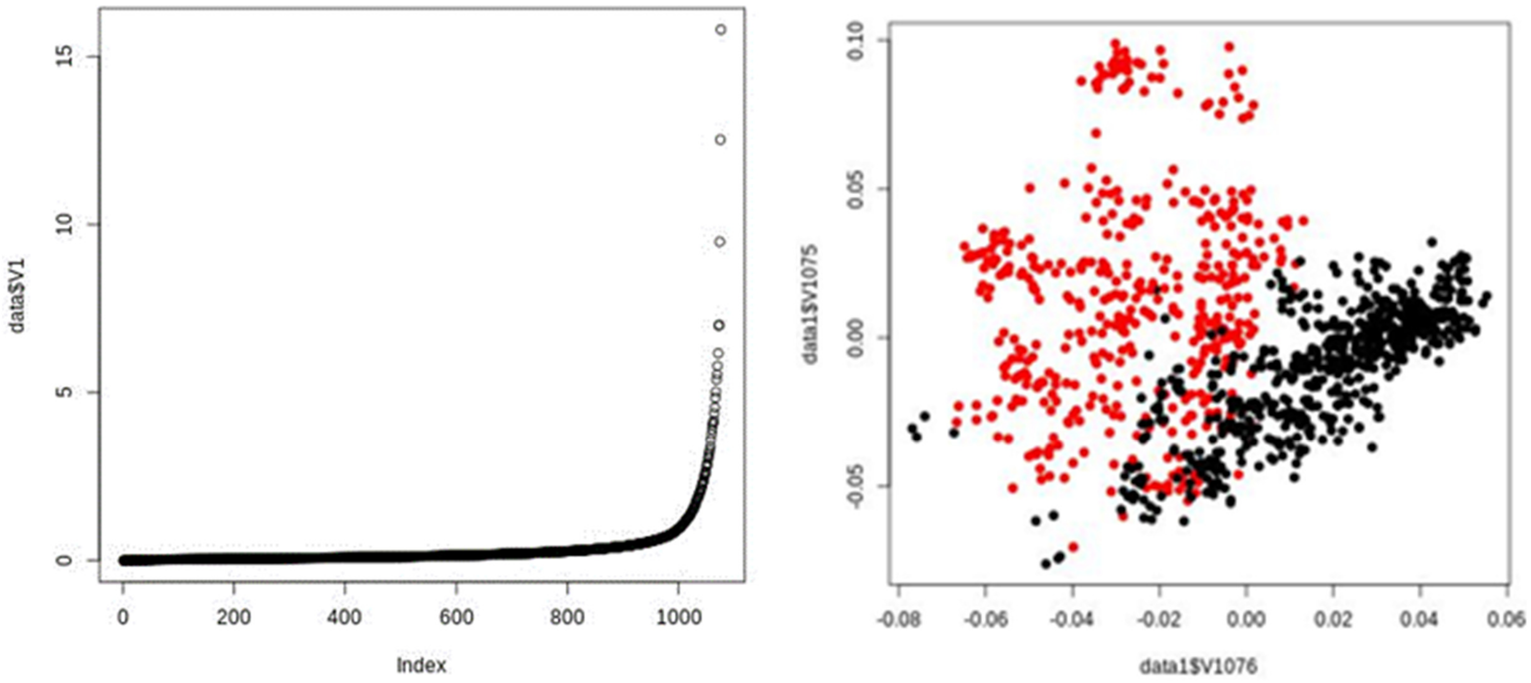

(a) (b)

Figure S12. PCA analysis in European sea bass. The last two components from the PCA analysis (batch 10 corresponds to red color and batch 13 corresponds to black color).
